# Parents’ experiences of family-based physical activity interventions: a systematic review and qualitative evidence synthesis

**DOI:** 10.1186/s12966-025-01778-9

**Published:** 2025-07-01

**Authors:** Carol Brennan, Evangeline Streight, Shishi Cheng, Ryan E. Rhodes

**Affiliations:** 1 Behavioural Medicine Laboratory, School of Exercise Science, Physical and Health Education, University of Victoria, Victoria, Canada; 2 Department of Sport, Physical Education and Health, Faculty of Arts and Social Sciences, Hong Kong Baptist University, Kowloon, Hong Kong

**Keywords:** Physical activity, Family, Parents, Qualitative synthesis, Thematic synthesis

## Abstract

**Background:**

Children and adolescents are at increased risk of adverse health consequences linked to physical inactivity. Parental support is positively correlated with children and adolescents’ physical activity (PA) levels. As a result, family-based interventions are acknowledged as an effective strategy for enhancing PA among this cohort. However, the effects of these interventions on child and adolescent PA are often inconsistent, with calls for more in-depth understanding of the contextual issues related to intervention implementation and parents’ experiences of interventions. The purpose of this review was to appraise and synthesize qualitative research regarding parents’ experiences of family-based PA interventions.

**Methods:**

Seven databases (MEDLINE, PsycINFO, SportDiscus, CINAHL, Web of Science, Scopus, and ProQuest) were searched for studies published from inception to January 2024 that included qualitative evaluative data of parents’ experiences of family-based PA interventions. The research quality of included studies was appraised using the Critical Appraisal Skills Programme. Qualitative data were extracted and thematically synthesized.

**Results:**

A total of 7,770 articles were screened, of which 82 independent studies were included in the final synthesis. Three analytic themes were generated. (1) The reasons why parents enrolled in family-based interventions and the perceived benefits for parents, children, and families; (2) Parents' perspectives on intervention components, including their satisfaction, coherence, and suggestions for improvement; (3) The social and environmental factors shaping parents’ intervention experiences and parental PA support. Findings show the benefits of PA, being a good role model and spending time together as motives for enrollment. Parents perceived child or adolescent’s PA confidence and overall well-being and family functioning improved. Parents presented mixed views about planning, goal setting, self-monitoring, intervention materials and resources, and delivery. Child or adolescents’ interest, social connections, financial constraints and availability of resources impacted parental engagement.

**Conclusions:**

This novel and comprehensive review offers practical recommendations to guide intervention development and inform future policy and practice such as: consider using co-design methods and social network analysis; promoting the benefits of PA on family functioning during recruitment; strengthening parents PA support identities; provide opportunities for social support for families post-intervention and educate coaches to create an environment of inclusivity and enjoyment.

**Trial registration:**

PROSPERO CRD42023421539.

**Supplementary Information:**

The online version contains supplementary material available at 10.1186/s12966-025-01778-9.

## Background

The health benefits of physical activity (PA) for all ages are widely accepted. For children and adolescents (from here on defined as youth), they are associated with improved physical, psychological, social, and cognitive health, including reduced adiposity, academic achievement and overall mental health [1–3]. According to the World Health Organization (WHO) youth should engage in an average of 60 mins of moderate-to-vigorous intensity, mostly aerobic, PA across the week to benefit their health [4]. However, despite these benefits, globally 81% of youth are not meeting the recommended PA guidelines [5], putting them at risk of the negative health consequences associated with physical inactivity [6]. Further, PA behavior during youth tracks into adulthood [7, 8], therefore, developing strategies to promote PA in this cohort is a public health priority.

A body of research examining PA interventions in youth reported how most interventions took place within the school setting [9, 10] with mixed findings of effectiveness on PA levels [11, 12]. However, youth spend a significant amount of their time within the care of their parents and are reported to be less active in time spent outside of school (e.g., at weekends or holidays) [13, 14] Furthermore, parents have been described as “gatekeepers” to PA during family time [15]. Parental support is an overarching term used to characterize several support behaviors for PA such as encouragement (e.g., offering praise, feedback and spectating), logistical support (e.g., provision of transport), co-activity (e.g., parents providing support through being active with their child), environmental support (e.g., provision of materials and equipment necessary to facilitate PA), and regulatory support (e.g., establishing rules and regulations around PA) [16, 17]. There is considerable evidence establishing that parental support for PA is positively correlated to youth PA [18–20]. Thus, there is a need to position parents as a central part of interventions promoting PA in youth [9, 17].

Family-based interventions are recognized as an advantageous route to improving PA behaviors [21], however systematic reviews of family-based interventions have reported inconsistency regarding the effectiveness of these interventions on youths’ PA [22–25]. This inconsistency may be due to variability in study design, diverse outcome measures, and methodological weaknesses [22–25]. Furthermore, most of these reviews [23–25] involved quantitative methods, thus offering limited insight into how or why the effects of family-based interventions differ. Although, Brown et al. [22], used a dual approach of meta-analysis and realist synthesis to enhance our understanding of the outcome patterns of family-based interventions with children aged 5–12 years. A realist synthesis is a recognized approach for explaining the relationship between the context in which an intervention is implemented, the mechanisms of how interventions work and the outcomes generated [26]. This provides decision-makers with a detailed understanding of why interventions work and how they can operate more effectively [26]. For example, Brown et al. [22], reported that when a combination of goal-setting and reinforcement strategies were used (i.e., context) they enhanced parents’ motivation to support changes in their child’s PA behavior (mechanism), which in turn contributed to an increase in child PA levels (outcome). However, only 28 of the 47 studies in the review provided sufficient information to describe the outcome patterns and while the authors sought supplementary papers such as process evaluation papers, due to time constraints they did not conduct iterative searches for related papers, thus restricting the ability to fully explain how and why each intervention worked [22]. Furthermore, this review did not identify whether any of the studies targeted families of low socio-economic position (SES) or include adolescents or populations with illness or disability. Thus, there is a need for a more in-depth focus on parents’ perspectives and experiences to identify factors that may increase engagement, reduce barriers to implementation and help tailor interventions for specific populations.

A qualitative evaluation of complex interventions is also recommended by the UK Medical Research Council [27] and the WHO [28] to answer questions beyond intervention effectiveness, and typically collected via qualitative surveys, exit interviews or focus groups [27, 28]. Evaluating interventions qualitatively provides a contextualized understanding of complexity of interventions, the challenges related to implementation and a deeper insight into participants experiences [29]. Whilst qualitative studies with parents experiences of family-based in interventions targeting PA in youth have been published, this literature has not yet been synthesized. A qualitative evidence synthesis (QES) enables a rich interpretation of the issues relating to intervention feasibility, acceptability and adherence from the perspective of the participants, and helps understand the influence of the intervention on different subgroups of people [30, 31]. Further, a QES assists researchers to “go beyond” individual study findings and generate insights that exceed the sum of their parts [32]. Thus, findings that may not be considered noteworthy within a single qualitative study can be identified and support the development of more powerful and insightful explanations [31–33]. Therefore, the aims of this systematic review and QES were (1) to identify, appraise and synthesize qualitative research regarding parents’ perspectives and experiences of family-based interventions targeting PA in youth and (2) provide recommendations to guide intervention development and inform future policy and practice promoting youths’ PA.

## Methods

This systematic review was prepared in accordance with the Preferred Reporting Items for Systematic reviews and Meta-Analysis (PRISMA) 2020 guidelines [34] (see Additional file 1) and the Enhancing Transparency in Reporting the Synthesis of Qualitative Research (ENTREQ) guidelines (see Additional file 2) [35]. A protocol for this review was registered with the International Prospective Register of Systematic reviews (PROSPERO) (registration number: #CRD42023421539).

### Search strategy

A systematic search was carried out in seven electronic databases (Medline, PsycINfO, Cumulative Index to Nursing and Allied Health Literature (CINAHL), Web of Science, SportDiscus, Scopus, and ProQuest. The searches were conducted on January 25th, 2024. Search terms were developed from initial literature scoping, consultation with a research librarian, and were piloted to ensure relevant studies were being included. The search strategy combined terms relating to family-based interventions, physical activity, parents, children and adolescents, youth, and qualitative or mixed methods research (see Additional file 3 for search terms). No date limits were applied, and results were restricted to those published in English, French and Chinese.

### Eligibility criteria

Studies of interest were defined as any family-based physical activity intervention that provided qualitative data around an exit/end of study/intervention follow-up. Studies were included if they had (1) a family unit within the intervention, consisting of at least one parent/guardian and a child under the age of 18 years; (2) delivered an intervention that involved altering the PA (e.g., exercise, play, sport) of a youth(s) with parental/guardian involvement; and (3) collected qualitative information from a parent/guardian after the intervention. Studies included published articles and dissertations from thesis work. All types of experimental study designs (e.g., pre-post, RCT, within-subject designs) were included. We excluded studies primarily based in schools or communities without a family component. Finally, studies were excluded if promoting change to a youth’s PA was not a primary outcome and/or did not include a post-intervention qualitative assessment.

### Study screening methods

All references were imported into EndNote X9 software for duplicate removal and for a manual search conducted by one reviewer (E.S.). The references were then imported into ASReview, an open-source machine learning framework for systematic reviews that facilitates the organization of references based on their relevance to the research topic [36]. The efficacy of the tool has been established by previous studies [36, 37]. Specifically, the software was trained using four references that met the inclusion criteria and four irrelevant references that were randomly suggested by the programme [38]. Using these references, the software prioritized all records from most relevant to least relevant, it continuously adapted by learning from the reviewer's decisions as relevant or irrelevant based on the inclusion criteria and adjusted the ranking of references accordingly. Locating all relevant records is extremely challenging, even for humans who typically overlook approximately 10% of relevant records [39]. Nonetheless, to minimize the risk of missing relevant papers in the screening process, guidelines provided by van Haastrecht et al. [38], were followed. One reviewer (E.S.) screened titles and abstracts until our pre-determined, data-driven stopping criterion of 55 consecutive irrelevant records was reached [38]. A second reviewer (H.Z.) independently reviewed all titles and abstracts that were excluded by the first researcher. A full-text examination of all remaining records was conducted by two reviewers (E.S. and H.Z.), and any disagreements were resolved through discussion with a third researcher (R.E.R.). Additionally, the first author screened the reference lists of included manuscripts and systematic reviews of family-based interventions [22, 24, 40] to identify other studies that may have been screened irrelevant or not included in the initial search.

### Data extraction and quality appraisal

General study characteristics (author, year, country of study, research aims, phenomenon of interest, participants, methodology, data collection, data analysis, intervention design, characteristics and outcomes) were extracted from the included studies, by one author (C.C.) using a modified data extraction form in Microsoft Excel and reviewed by a second author (C.B.). The methodological quality of the included studies was assessed using the Critical Appraisal Skills Programme (CASP) checklist [41]. The CASP is a well-recognized checklist for assessing quality in qualitative studies and has been previously used to assess qualitative studies which seek to understand experiences of interventions in PA [42, 43]. The appraisal was conducted by two independent reviewers (C.B. and R.E.R.), and any disagreements were resolved through discussion between them.

### Analysis and synthesis

Data were analysed using a thematic synthesis [44]. This method of analysis was chosen as it enabled us to remain close to the original context of the primary qualitative studies and draw conclusions about parents’ experiences across intervention types to develop analytic themes which go beyond the explanations reported in the primary studies [44]. Extracted data was managed and coded using Microsoft Excel software. Firstly, one author (C.B., an experienced qualitative researcher) conducted an inductive line-by-line coding of the included data into free codes which portrayed the meaning and context of each line of text. We did not distinguish between study type (e.g., age group, illness or disability) at this stage as our focus was on gathering rich explanations of the meaning portrayed in the data. All codes and the associated text were second checked by E.S., who acted as a critical friend by reviewing, questioning, and discussing the codes to enhance consistency and trustworthiness [45]. The next stage involved C.B. organizing and grouping of the free codes into descriptive themes and further discussion with E.S. Finally, C.B. generated analytic themes that accounted for patterns that became apparent across the included studies. This process was repeated until the analytic themes adequately encompassed the descriptive themes and addressed the review question. The analytic themes were discussed with the wider review team (C.B., E.S., C.C. and R.E.R.), who acted as critical friends by engaging in reflective dialogue, challenging interpretations, and contributing to the refinement of the themes during both the analysis and write-up stages.

## Results

### Study selection

**Fig. 1:**
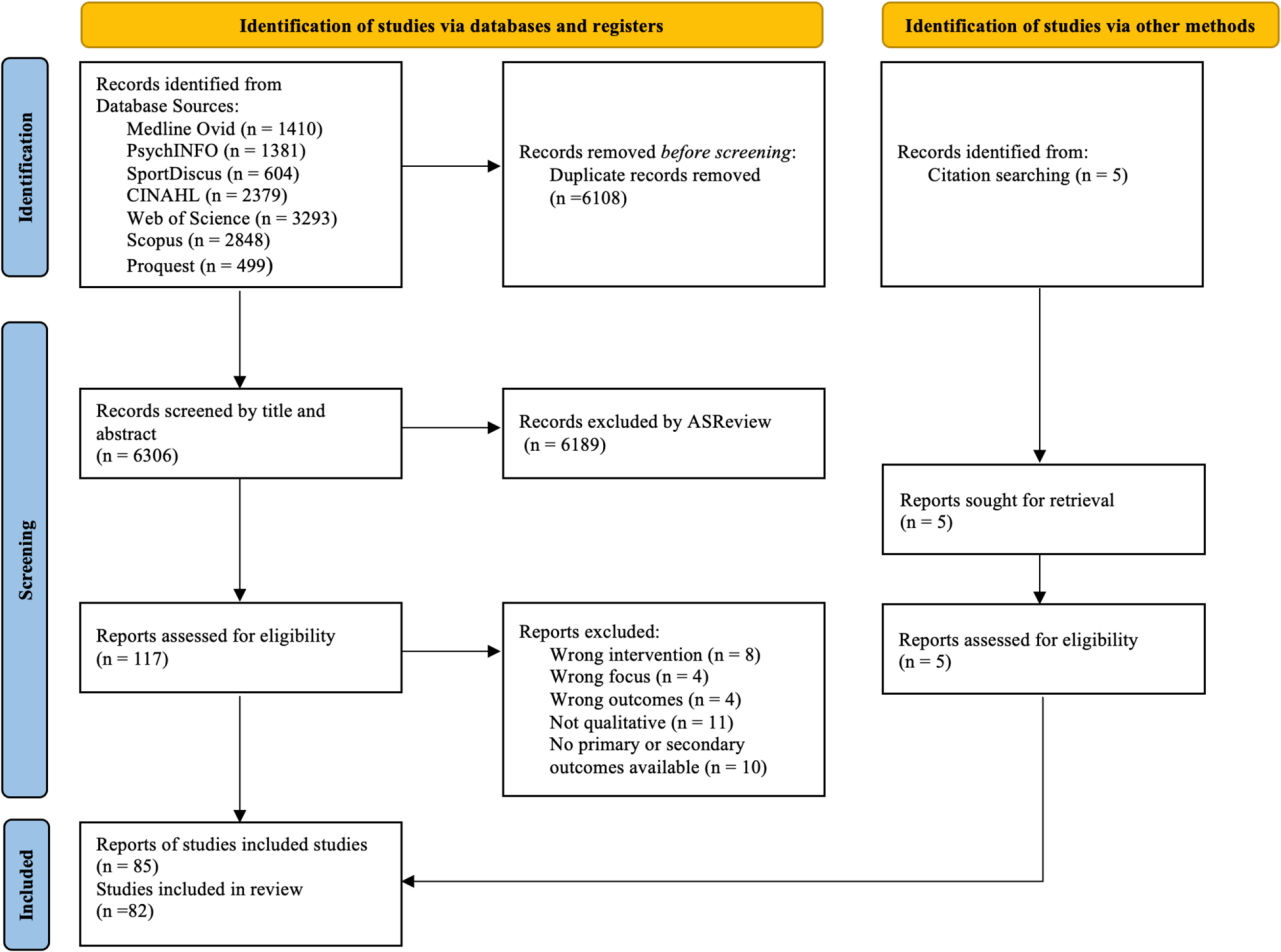
PRISMA 2020 flow diagram of included studies [34]

The search identified 12,414 records. Following the removal of duplicates, 7,770 titles/abstracts were screened. Of these, 117 full text articles were assessed for eligibility with a citation search providing an additional five records. This resulted in 85 reports representing 82 independent studies included in the review (Fig. 1).

### Characteristics of included studies

A summary of the included studies is presented in Table 1 (see Additional file 4) for full data extraction of each study). The studies were published between 2001 and 2024. Studies were conducted in the USA (k = 41), the UK (k = 13), Australia/New Zealand (k = 10), Canada (k = 8), Europe (k = 8) and Asia (k = 2). Twenty-seven studies focused on youth with overweight/obesity, three on youth with autism, while seven studies included youth with intellectual and/or physical disabilities (e.g., visual impairments, cystic fibrosis). Eight studies included people of low socioeconomic status (SES). Most studies (k = 63) were family-based, seven were parent–child only, eight were mother–child only, and four were father-child only. Fourteen studies were of children ages 3–5 years, 46 of children ages 6–12 years, 13 of adolescents ages 13–18 years and nine were mixed groups of youth ages 6–18 years. Many studies used semi-structured interviews for data collection (k = 48), 25 studies used focus groups, while four studies used both interviews and focus groups. Methods of analysis included thematic analysis (k = 26), content analysis (k = 15), grounded theory (k = 7), while 32 studies did not state the method of analysis used.

**Table 1:** Overall study characteristics

	Number of studies K (%)
Geographic location	
Australia	9 (11)
Canada	8 (10)
China	1(1)
Denmark	1(1)
Ireland	1(1)
Lithuania	1(1)
New Zealand	1(1)
Norway	1(1)
Sweeden	4 (5)
Turkey	1(1)
UK	13 (16)
North America	41 (50)
Population type	
Non targeted/ general intervention	38 (46)
Low-SES^a^	8 (10)
Children with overweight/obesity^a^	27 (33)
Children with autism	3 (4)
Children with intellectual and/or developmental disabilities	2 (2)
Children with type I diabetes	1 (1)
Children with type II diabetes	1 (1)
Children with cystic fibrosis	1 (1)
Children with visual impairments	1 (1)
Children with a mobility disability	1 (1)
Intervention design	
Family- based	63 (76)
Parent—child	7 (9)
Mother—child	8 (10)
Father—child	4 (5)
Age groups of youth in the intervention	
3—5 years old	14 (17)
6—12 years old	46 (56)
13—18 years old	13 (16)
Mixed group of youth 6–18 years	9 (11)
Participant groups in exit interview/focus group/questionnaire	
Mothers only	8 (10)
Fathers only	5 (6)
Parents (mothers and fathers)^b^	69 (82)
Unclear (Family members: no. of parents and children not identified)	2 (2)
Methods	
Interviews	48 (57)
Focus Groups^b^	25 (30)
Interviews and focus groups	8 (10)
Qualitative surveys	3 (3)
Analysis	
Thematic analysis	26 (31)
Content analysis	15 (18)
Grounded theory	7 (8)
Phenomenological analysis	2 (2)
Content and thematic analysis	1 (1)
Framework analysis	1(1)
Hermeneutic analysis	1 (1)
Not mentioned	32 (38)

^a^1 study targeted low SES and children with overweight/obesity (Cason-Wilkerson)

^b^1 study used the same participants for both reports (Cason-Wilkerson)

### Assessment of quality and confidence in review findings

Overall, studies were of mixed quality. Eleven studies covered the 10 items on the CASP checklist [41], 54 covered between seven and nine items and 20 covered between three and six items (see Additional file 5). The main items not addressed across the studies were the consideration of the relationship between the researchers and the participants (k = 72), whether the research design was appropriate to address the aims of the research (k = 24), and reporting of data analysis methods sufficiently (k = 21). Studies scored well on clearly stating the aims of the study (k = 76) and the appropriate selection of qualitative methodologies to address the study’s aims (k = 66).

### Qualitative evidence synthesis findings

Nine descriptive themes were developed in the analysis. From these four analytical themes were generated encompassing the factors that facilitate or hinder parents’ participation in family-based PA interventions. An overview of the findings and the contributions of the studies are presented in Table 2 and summarized below.

**Table 2:** Summary of findings and contributing studies

Analytical Themes	Descriptive Themes	Contributing studies^a^	Studies with ages 3–5 years	Studies with ages 6–12 years	Studies with ages 13–18 years	Studies with ages 5–18 years	Studies of youth with overweight or obesity	Studies of youth with illness or disability
			K** (%)**	K** (%)**	K** (%)**	K** (%)**	K** (%)**	K** (%)**
The reasons why parents enrolled in family-based interventions		12, 14,17, 23, 24, 26, 27, 33, 35, 36, 38, 41, 51, 53, 56, 57, 61, 67, 68, 70, 75, 77, 78, 79, 83	3 (12)	13 (52)	5 (20)	4 (16)	6 (24)	2 (8)
The perceived benefits of taking part for parents, children and families	Taking part improved parents’ knowledge and confidence about PA and parental PA support	1, 2, 5, 6, 8, 9, 10, 11, 12, 13, 14, 16, 20, 22, 23, 24, 25, 26, 29, 30, 34, 35, 37, 38, 40, 41, 45, 46, 47, 48, 56, 57, 59, 61, 65, 66, 67, 68, 72, 74, 75, 77, 78, 81, 83	9 (20)	25 (56)	5 (11)	6 (13)	14 (31)	5 (11)
	The intervention increased child’s PA skills and confidence and enhanced their wellbeing	3, 4, 6, 11, 20, 22, 23, 24, 26, 27, 32, 37, 41, 42, 43, 45, 46, 49, 50, 55, 57, 59, 61, 62, 63, 65, 67, 72, 75, 80, 81, 84, 85	4 (12)	20 (61)	5 (15)	4 (12)	8 (24)	6 (33)
	Participation resulted in greater family activity levels, and enriched family functioning and parent–child relationships	1, 3, 4, 5, 7, 9, 11, 12, 13, 16, 17, 20, 21, 22, 24, 26, 29, 31, 34, 37, 38, 41, 42, 43, 44, 45, 46, 54, 55, 57, 59, 61, 63, 64, 65, 66, 67, 72, 73, 74, 76, 77, 78, 81	15 (34)	19 (43)	6 (14)	4 (9)	9 (21)	5 (11)
Parents' perspectives on the intervention components, including their satisfaction, coherence, and suggestions for improvement	Mixed views about planning, goal setting, self-monitoring and intervention materials and resources	6, 7, 8, 9, 11, 14, 19, 25, 26, 29, 39, 41, 43, 47, 57, 58, 65, 67, 69, 71, 74, 76, 79, 81, 82, 84	4 (16)	12 (46)	5 (19)	5 (19)	5 (19)	4 (16)
	Intervention mode, setting, source and style of delivery impacted engagement in the intervention	6, 7, 9, 11, 14, 16, 17, 19, 21,23, 24, 25, 26, 27, 29, 30, 32, 34, 35, 36, 40, 41, 42, 43, 44, 46, 47, 48, 49, 51, 52, 56, 57, 59, 61, 63, 64, 65, 66, 69, 70, 72, 75, 78, 79, 83, 84	8 (17)	23 (49)	10 (21)	6 (13)	15 (32)	5 (11)
	Mostly a fun, engaging and worthwhile experience with a need for social connection post-intervention	1, 6, 8, 10, 11, 12, 15, 16, 17, 19, 21, 22, 24, 25, 26, 27, 28, 30, 31, 33, 34, 37, 41, 44, 45, 46, 47, 48, 49, 51, 52, 53, 57, 58, 59, 60, 61, 63, 65, 66, 67, 68, 69, 70, 71, 72, 73, 75, 77, 79, 81, 82, 83 85	12 (22)	31 (57)	6 (11)	5 (10)	14 (26)	7 (13)
The social and environmental factors shaping parents’ intervention experiences and parental support for PA	Family interest in PA and other social influences affect parents’ intentions towards the intervention and parental support for PA	1, 2, 4, 5, 6, 9, 10, 18, 21, 23, 26, 29, 30, 32, 33, 34, 35, 36, 37, 38, 41, 43, 44, 45, 46, 48, 49, 55, 57, 58, 61, 63, 65, 66, 67, 70, 72, 74, 78, 79, 81, 82, 83	8 (19)	22 (51)	6 (14)	7 (16)	11 (26)	4 (9)
	Competing priorities, financial constraints and safety concerns influences parental PA and how parents support their child to be active	1, 5, 8, 10, 11, 12, 13, 14, 17, 18, 20, 21, 22, 25, 26, 29, 31, 32, 33, 36, 37, 38, 41, 43, 44, 45, 47, 48, 51, 58, 60, 61, 62, 63, 65, 67, 70, 77, 79, 80, 81, 82, 83	7 (16)	25 (58)	6 (14)	5 (12)	12 (27)	6 (14)

^a^list of contributing studies provided in Additional file 6

#### The reasons why parents enrolled in family-based interventions

This analytical theme describes how the motivating factors for parents’ engagement in family PA interventions are centered around a desire to improve the health and wellbeing of their entire family coupled with the opportunity to spend time together. In several studies (K = 25), parents discussed why they enrolled in the intervention. These reasons were closely aligned with their perceptions of how the intervention would address their family’s needs. Parents expressed a strong desire to make lifestyle changes that would improve the quality of life for the whole family. They recognized and understood the immediate and long-term physical and mental health benefits of being physically active and more broadly living a healthier lifestyle. In some studies parents were motivated by concerns for their child’s health, particularly if their child had a chronic disease or was overweight/obese. For example, a parent in an obesity prevention intervention shared: “*Because I want my daughter to be healthier... my daughter is overweight. And her lab tests showed fatty liver... so I want her to learn how to eat better and do physical activity.”* [46] (Parent, child aged 6 - 12 years).

Parents described their desires to act as a ‘good’ role model for their children/adolescents and that by making changes to their own PA and lifestyle behaviors they believed it would encourage their children/adolescents to do the same, as shared by a parent *“I think it starts with home. You gotta be able to be active yourself so their kids can see that... you’re trying to get them involved with what they’re doing because if you’re not doing it, they’re not gonna do it.”* [47] (Parent, child in mixed age group, 6 - 18 years) Finally, parents discussed how taking part in a family-based intervention would provide them with an opportunity to spend more time together as a family, improve their relationship with their child(ren), form bonds and work on their health goals together, for example a father shared:

*“Any time you’re able to spend time with them, where they are enjoying it, it is always a good thing...It gives an extra few hours a week that you are able to be with them and have a good time with them.”* [48] (Parent, child aged 6 - 12 years)

#### The perceived benefits of taking part for parents, children, and families

This analytical theme illustrates how taking part in the intervention improved parents’ knowledge and confidence around PA and how to support their child(ren) to be active. Additionally, parents shared how their child’s skills and confidence to be physically active increased and their wellbeing improved. Overall, participation led to increased family activity levels, enhanced family functioning, and strengthened parent–child relationships. This theme encompasses three descriptive themes, described below.

##### Taking part improved parents’ knowledge and confidence for PA and parental PA support

Parents described how the intervention increased their knowledge and understanding of the benefits of PA, and awareness of available resources in their home and local area, thus strengthening intentions to support their child(ren) to be active (K = 45). Feedback from interventions that provided physical literacy training for parents highlighted an increase in awareness of the types of activities their child(ren) could do in the home environment with minimal equipment. As shared by a parent:

*“Sometimes you can get stuck in thinking, “I can’t do much at home” or “I have to go to a gym or rec center to do these kind of things”. But the workshop just kinda opened my eyes for other activities that we can do at home very easily and that kind of inspired me to find other similar things that we can do.”* [49] (Parent, child aged 6 - 12 years)

It is noteworthy that some parents of children with disabilities were unaware of the types of activities that their child could engage in or were uncertain of their child’s ability to be physically active, thus these interventions played an important role in educating parents around PA for their child. For example, a parent described how an intervention for children with visual impairment *“[got] things started because I never thought that PA was something that visually impaired children did.”* [50] (Parent, child aged 6—12 years).

Additionally, in interventions that contained lifestyle components such as diet, sleep quality, and reducing screen time, parents reported how they embraced a combination of healthy lifestyle behaviors for themselves and their family members. They emphasized how being physically active had a positive impact on other lifestyle behaviors. This is an important finding for families at-risk of the negative health consequences associated with physical inactivity. An intervention to improve healthy behaviors in American Indian families with young children provides an example from a parent:

“*I learned a lot and this program helped keep me motivated to stay eating healthy and be more physically active. I now eat veggies every day and I cook dinner more and watch a lot less TV and actually we don’t even watch TV more than 2 times a week.'* [51] (Parent, child aged 3 - 5 years)

Alongside increased knowledge around PA and healthy lifestyle behaviors parents expressed how the intervention increased their awareness of the important role parents play in their child's PA and lifestyle behaviors. They described how the strategies and skills learned enhanced their confidence and ability to support their child to be active. A parent in a lifestyle intervention for youth with obesity shared, *“Empowered. Yeah, definitely. You feel like you have more knowledge to…work everything out, you know?… Whether it’s with the nutrition or exercise … You feel like you have some more tools to kind of deal with things.”* [52] (Parent, child in mixed age-group, 6—18 years) Furthermore*,* parental involvement in the intervention provided parents an opportunity to spend time with their child(ren) and helped them see how their child enjoyed being active and learning new skills. This was particularly important for parents of children with disabilities, as their involvement offered reassurance in their child’s abilities, thus increasing their confidence towards continuing to support their child post-intervention. As one parent shared, “*But mostly, it has helped me become less protective of my son. Now I can see he can do things on his own. So a part of this has been me letting go, after seeing he can do things by* himself’ [53] (Parent, child in mixed age-group, 6—18 years).

##### The intervention increased child’s PA skills and confidence and enhanced their wellbeing

Across many studies (K = 33) parents described how the intervention’s activities (e.g., soccer, hockey, rock climbing, swimming, kayaking) helped improved their child’s skills, confidence, and enjoyment in being active, with the most notable differences reported in interventions with younger children and those with disability or illness. A parent in an intervention for overweight/obese children and youth with intellectual disability shared “*Before this program my son couldn’t walk from here out to the road, and now he can run. Now he can run!”* [54] (Parent, child in mixed age-group, 6—18 years) In some studies, the child became the agent of change for the family by encouraging parents and other family members to partake in the intervention activities that involved outdoor play, practicing skills, and family walks or bike rides. As one parent described “*He (child) has probably made us more aware I think of what we need to do to do better”* [55] (Parent, child aged 6—12 years).

Parents discussed how the intervention made a positive contribution towards their child’s wellbeing, by improving their child’s independence, confidence, and interpersonal skills. This resulted in their child displaying an increased willingness to interact with others and try new things, including extracurricular activities that they would not have engaged in prior to partaking in the intervention. A parent explained, “*Before the program my child was signed up for nothing, and now she’s signed up for 3 things.... So she’s doing karate, yoga, and skating.”* [56] (Parent, child aged 6—12 years)Additionally, engagement in the intervention enhanced parental perceptions of improvements in their child’s self-concept and self-esteem, which subsequently had a positive influence on other factors such as resilience in challenging situations, increased energy levels and school attendance. Indeed, this was pertinent in at-risk children from a minority group who took part in a free, outdoor, activity-based, parenting intervention delivered over multiple sessions to vulnerable families. As shared by a parent:

*"The kayaking helps my child to connect with his culture and be proud to be Aboriginal. He won an award at school ... he wasn't attending, he was an at-risk student, but the program changed all that. He never would have felt so proud before...he is more confident, and he couldn't do that without the kayaking program."* [57] (Parent, child aged 6 - 12 years)

##### Participation resulted in greater family activity levels, and enriched family functioning and parent–child relationships

In many studies (K = 44) parents described how their entire family became more active together and family functioning and parent–child relationships improved since enrolling in the intervention. For some physical activity became a regular part of their family’s routines, as shared by one parent, *“It actually changed family habits, thinking, perceptions.”* [58] (Parent, child aged 6—12 years) It was important for most parents to find activities that were suitable for the entire family while others shared how their whole family had a positive and sustained attitude change towards PA. A parent in a community-based intervention described, “*We’ve been exercising a lot more, five times a week now, every week. It’s been like three months now. We’ve been doing that since we ended the program. It’s been pretty good.”* [59] (Parent, child aged 3—5 years) Some parents attributed the increased family activity levels to their beliefs that it was easier to encourage their children to be active when the parents were also involved. As illustrated by a parent *“The children feel more motivated with mom and dad, so the whole family is together. [When] we all go for a walk, swimming, or doing a sport, they feel more motivated.”* [60] (Parent, child aged 6—12 years).

Parents explained how taking part in the intervention helped improve overall family cohesion and organization. Parents shared how by spending more time together in the intervention they had fun, became closer and more united as a family, and tried activities that they might otherwise not have engaged in. As illustrated by a parent,

“*I think that this brought to our family, it taught us a lot about unity, about team. And not only in our comfort zone, but we got a little bit out of our comfort zone. And it brought us closer as a family, we did a lot of things as a family, but with FIT4YES, we had a lot of fun, things that I probably wouldn't have planned on our own” * [61] (Parent, child aged 6 - 12 years)

Additionally, parents described how the inherent nature of family physical activity meant that all external distractions (e.g., mobile phones, TVs) were removed so the focus was solely on themselves and their child(ren). This resulted in long-term improvement in the quality of communication, parenting, and time they were spending together as a family. As illustrated by a parent in a family-based intervention which explored planning as a strategy to increase PA “*You just interact in a different way than if you are like, say driving… being together doing an activity is a different dynamic which is fun that you don't really do in any other way." * [62] (Parent, child aged 6—12 years).

Parents discussed how individual relationships improved with their child, particularly for interventions that were parent–child focused e.g., father-daughter or mother-daughter and where there was a dedicated parent–child PA or sports component. These interventions provided the parent–child dyad with an opportunity to have fun, spend quality time together and engage in one-on-one bonding. A parent in a father-daughter PA intervention explained how his relationship with his daughter developed *“It’s like the connection between us, that we used to hug before, and we hug now, it feels like there’s more glue connecting us* [63] (Parent, child aged 6—12 years).

#### Parents'perspectives on the intervention components, including their satisfaction, coherence, and suggestions for improvement

In this theme, parents highlighted how the intervention's content, materials, and resources either enhanced or limited their ability to adhere to the intervention and support their child(ren) in maintaining PA levels both during the intervention and in the long term. Furthermore, parents described how the interventions’ mode, setting, source and style of delivery affected their engagement in the intervention. Finally, parents shared their perspectives on intervention coherence, satisfaction and suggestions for improvement. This analytical theme is supported by three descriptive themes discussed below.

##### Mixed views about planning, goal setting, self-monitoring and intervention materials and resources

Across several studies (K = 26) parents shared their perspectives on the intervention content, materials and resources that either facilitated or hindered their ability to adhere to the intervention’s objectives and ultimately support their child(ren) to be active both in the short-term and post intervention. The types of intervention content most frequently reported by parents were planning, goal setting, self- monitoring, monitoring, and prompt/cues. Parents described how they actively worked together with their child(ren) to plan their activities for the week ahead, this fostered a sense of shared commitment to their goals, thus increasing the likelihood of success, especially where the goals were viewed as achievable and gradually increased in difficulty or intensity each week. To illustrate, a parent in an intervention discussed:

“*They [facilitators] told us to set realistic goals. You know you cannot expect to start off at, say, number three [on 1‐10 scale] and expect to be number ten by the end. Being aware that something was not right in the first place and then you can build from it from there. [G3]”* [64] (Parent, child aged 3 - 5 years)

Notably, while parents acknowledged the significance and advantages of planning and goal setting, others highlighted challenges in effectively translating these approaches into the practice of family physical activity, as expressed by a parent, *“I definitely think it helped but not as far as execution”* [62]*.* (Parent, child aged 6 - 12 years).

Parents reported how using intervention resources such as workbooks, pedometers, or activity trackers to complete daily logs of activity levels provided both parents and children with insight into their PA and health related behaviors (e.g., diet and sleep), enabled them to self-monitor their progress, and boosted motivation to continue working towards their goals. However, in some cases parents found self-monitoring burdensome and challenging to complete, as shared by one parent “*Excellent idea. Realistically, difficult to do.”* [65] (Parent, child aged 6 - 12 years) Parents commented how their children derived a sense of pride and achievement in checking their progress, a mother in an intervention shared “*They’d come and tell me they’d done this many runs. It really worked for them. It was really a motivator.’* [66] (Parent, child aged 6 - 12 years) Parents mostly discussed how their children/adolescents enjoyed using the pedometers to track their step counts, with only a few reporting incidents of ‘too much competition’ between the children or an over reliance on the pedometer to be active. For example, a mother described how her adolescent daughter was “*devastated, she wasn’t walking anywhere that day because it wouldn’t count for anything.”* [67] (Parent, child aged 13 - 18 years) The use of accelerometers reported mixed findings, with some parents describing that their child was happy to wear it while other children found it more challenging. One parent discussed *“My son, yeah. He wore it and then he was complaining like it was too tight sometimes.”* [68] (Parent, child aged 6—12 years) When it came to intervention handouts and workbooks there were mixed findings, occasionally within the same intervention with some parents citing a preference for online versions while others preferred printed versions. A parent explained: *“I’m an old-fashioned person in that way […] I like printed things because then you can go back and you have that. I read it more carefully when I have it printed.”* [69] (Parent, child aged 6 - 12 years).

##### Intervention mode, setting, source and style of delivery impacted engagement in the intervention

Intervention modes of delivery included face-to-face sessions, printed materials, video analysis, video conferencing, mobile apps and websites (K = 47). mHealth applications were considered acceptable for parents in our review due to their accessibility and ease of use. Interestingly, in a study by Ha et al., [70] a father cited a preference for a combination of online and face-to-face modes of delivery *“If there are future opportunities to conduct related activities, I believe the learning format can be alternated between face-to-face and online classes.”* (Parent, child aged 6 - 12 years).

The location, timing, frequency and length of face-to-face sessions were sometimes barriers to attendance for parents, although parents appreciated the opportunities face-to-face sessions provided them regarding meeting other parents and spending time with their child(ren). As shared by a parent:

“*You start the program at the beginning you’re with your kids then your kids leave and you get this great, adult interaction while your kids get to go hang out with friends, and I loved the way it’s set up. It’s set up to be family time, it’s an adult time and then family time again. Something about that was really special”* [59] (Parent, child aged 3 - 5 years)

Schools and local community centers were perceived as suitable locations for the intervention setting, primarily due to location, safety concerns, ready-made social networks, and parents desire for greater links between the school and parents when it came to promoting PA and healthy lifestyles, as a father explained, *‘it’s good to have it reinforced I think from somebody other than your parents, sometimes if your teacher says it, it’s true!’* [71] (Parent, child aged 6 - 12 years) Parents also cited the important contribution age-appropriate and adapted facilities made towards their child’s confidence for PA as cited by a parent in an intervention for children with visual impairment: *“he is on an even ground [in] an area where he can excel and feel really proud and do well.”* [50] (Parent, child aged 6 - 12 years). Overall parents described intervention facilitators, guest speakers, experts and counsellors as positive, enthusiastic, knowledgeable and approachable. Younger facilitators for children or facilitators of the same cultural background as the participants were perceived as relatable and understanding of the challenges that parents face. A parent in a culturally tailored intervention for Māori and Pacific Islander families shared *“They can relate to us, having Polynesian teachers and mentors helps us and motivates us."* [72] (Parent, child aged 6 - 12 years).

Many parents viewed the intervention delivery style as family-focused, warm and non-judgmental. Providing autonomy support to parents was important to them in interventions that offered tips for parenting practices and communication styles, and as a result this approach encouraged parents to provide autonomy support to their children. A parent shared:

“*It was constantly stressed that we’re not telling you what to do...we’re just showing you, giving you bits of information so that you can make choices.... As a parent, that’s exactly what I try and do with my daughter.”* [73] (Parent, child aged 6 - 12 years)

##### Mostly a fun, engaging and worthwhile experience with a need for social connection post-intervention

Overall, most parents described the intervention as beneficial, flexible and easy to implement, while presenting some suggestions for improvements (K = 54). Many parents reported how partaking in the intervention was an enjoyable and worthwhile experience, with parents of children with overweight/obesity sharing they would enroll again if the intervention were available in the future:

“*My final perception is I would do it again and I’m hoping it will be back because like I said, I do have my son who needs help. I think it’s a great program. I think the effort that you all put in it, um — it was just such a good program for the kids. It was positive. I appreciated that the kids were in a positive environment, you know”* [74] (Parent, child aged 6 - 12 years)

Additionally, these parents described how their children sometimes struggled to be included in activities with their peers, a challenge that tailored interventions were able to address. A parent in an intervention to support parents to facilitate participation in physical activity for children and youth with disabilities explained, *‘At home, we don’t often get invited to things with other children, but here, we are included in everything’* [53] (Parent, child in mixed age-group, 6—18 years).

Parents expressed a range of views regarding their ability to understand and apply intervention content, materials and resources, oftentimes within the same study. Most parents described how they and their child(ren) found the intervention topics fun, interesting and engaging. Many shared how it was easy to apply the interventions’ learnings at home particularly if they were provided materials (e.g., skipping ropes, balls, racquets) or tips on how to use non-expensive items from around the home (e.g., plastic bottles, towels) to create fun games or obstacle courses. A mother in a Zoom‐delivered intervention to increase the physical activity level in children with autism spectrum disorder shared:

*"We could easily implement the activities. So we did not have any difficulties while applying it. I think they are extremely easy-to-apply activities. The activities were already interesting, it wasn't too hard to get my daughter used to it and she had a lot of fun, of course we had fun with her."* [53] (Parent, child in mixed age-group, 6 - 18 years)

On the other hand, in some studies, parents described an inability to apply the knowledge gained or use the intervention materials or resources. A few of these parents requested more concrete strategies (e.g., practical tips, feedback) to assist them enact the learnings, as shared by a mother in an overweight and obesity study shared “*We are more aware of what we need to do, but... we are missing that ‘how’*.” [75] (Parent, child aged 6 - 12 years).

There were some notable requests for improvements from parents, namely more time allocated to physical activities, less time spent on educational only activities for children and adults, greater child participation in the intervention (either with or without parents), and additional follow-up sessions for parents and children to assist with sustaining the behavioral changes accomplished during the intervention period. One parent explained,

*“to have someone like that [from the program] calling, you know, contacting you, asking how you’ve been, what you’ve been doing, what your goals are for the next month, I think that would just reinforce the message, and it wouldn’t take a lot of time.”* [76] (Parent, child aged 6 - 12 years).

A recurring theme amongst parents was the need for further opportunities for parents and children/ adolescents to connect with other families post-intervention (e.g., through Facebook or WhatsApp groups). For parents of children with overweight/obesity, illness, or disability, providing opportunities for their child(ren) to continue social connections is particularly important for developing friendships and fostering a sense of belonging with other children who share similar experiences and challenges, as illustrated by a mother in one intervention *“If a peer group similar to this could be made for kids…. They’d probably all go to different schools, but they’d have the exact same issues and they won’t judge”* [77] (Parent, child aged 6 - 12 years).

#### The social and environmental factors shaping parents’ intervention experiences and parental support for PA

This analytical theme illustrates how parents' intentions regarding the intervention and their support for PA are affected by their child(ren)'s interest in PA and other social influences (e.g., partners, grandparents). Additionally, parents shared how competing priorities, financial constraints and safety concerns impact both parental PA and how they support their child(ren) to be active. This analytical theme is underpinned by two descriptive themes described below.

##### Family interest in PA and other social influences affected parents’ intentions towards the intervention and parental support for PA

In numerous studies (K = 43) there was a reciprocal relationship between parents engagement in the intervention and their perception of their families interest in taking part. This stems from parental perceptions of their families enjoyment in the intervention and willingness to participate thus when family members did not want to take part, it was challenging for the parents to remain interested, as a parent discussed *“Definitely less motivated for me when she is not engaged and interested at all.”* [49]*.* (Parent, child aged 6 - 12 years). Some parents also described difficulties in maintaining their families interest in the intervention, reporting how they 'lost interest' or became bored, consequently making it challenging for parents to complete the required tasks of the intervention. As shared by a father in home-based exercise program for children with cystic fibrosis ‘‘*I think that.. she is just little and at first she was very excited about it. Now it is becoming a chore to do’’ * [78]*.* (Parent, child in mixed age-group, 6 - 18 years).

Parents discussed how sharing experiences with others in the intervention created a sense of shared understanding and belonging. Parents expressed relief upon hearing that others had similar challenges and difficulties. As explained by parents in a family-based healthy lifestyle intervention for adolescents with overweight/obesity, *“We’ve all realized that we’re in the same boat, when we go home, we’re all fighting the same battles. So, it’s been really really good to know that we’re not alone”* [79] (Parent, child aged 13 - 18 years). Indeed, in some interventions parents created their own personal support group so that they could continue to connect with each other after the intervention. As shared by a parent in a family-based lifestyle intervention for children with overweight and obesity.

*“We started a thing online through Facebook, we sort of just join our own personal group to keep helping each other, and you know, we can add whatever we want to, and sort of check in on each other to see how we’re going, and that has helped”* [76] (Parent, child aged 6 - 12 years).

Parents described how support from other family members (e.g. partners, grandparents, other children) shaped their experiences of the intervention. For example, family members provided encouragement for parents who increased their own activity levels or tried to make healthy lifestyle changes (e.g., improved diet). A parent shared *“Very supportive, they did provide support. Cheering me up to do it, that we should do it for health’s sake, so the child would be better”* [46] (Parent, child aged 6 - 12 years). Occasionally, family members' lack of interest in or understanding about the intervention provided challenges for the participating parent, mostly when it came to implementing changes in the home environment (e.g., introducing new rules and regulations around PA and diet). This was mainly relevant for parents who were separated or divorced, and the children spent their time between two different households. A parent shared their perspective, *“I feel a bit lost…we have a split relationship with the children. One week at our house, one week at their father’s house. And [the father has] only come to one session…he didn’t really care about this….”* [52] (Parent, child in mixed age-group, 6 - 18 years).

##### Competing priorities, financial constraints and safety concerns influenced parental PA and how they support their child to be active

In many studies (K = 43) parents described how competing priorities such as work schedules, extracurricular activities for children and general responsibilities (e.g., food shopping, household chores), presented challenges for parents regarding intervention attendance, completing the intervention tasks or goals, or scheduling time for the family to be active together. A parent shared their experience, “*Time, time, time, time. It’s just I mean I find that’s the biggest barrier is finding time”* [62]*.* (Parent, child aged 6 - 12 years). Furthermore, a number of parents, mostly mothers, reported difficulties regarding their own PA, sharing that by the time they organized and spectated at their child(ren)’s activities or sports, they had no time or energy to be active themselves. For example, one mother described:

*One of the biggest challenges is now [my children] have something every single evening, and we are literally flying from where I pick them up at their after-school program, home, eat dinner, and then out to something for them. They continue to stay active. . . . But that forces me to go and be sedentary most of the time, at their things, watching them be part of those fun activities*.” [56] (Parent, child aged 6 - 12 years).

The weather, safety concerns, financial constraints, and availability of recourses in the community shaped parents' decision making around PA. While parents were aware of their child’s interest in PA and the resources available to them in their local area, some found it difficult to find activities that were suitable their needs or budget. A parent shared:

*We don’t have it easy financially now...she likes activities...she is very interested in gymnastics, and she likes to swim...she likes to dance. Yes, but we can’t give her much opportunity [...] I want to enroll her in the gymnastic courses, but when I think about the money. no, I just explain to her, well she couldn’t attend the course.”* [69] (Parent, child aged 6 - 12 years).

For families who lived in areas of social deprivation, anti-social behavior meant they were reluctant to encourage their children to be active outdoors or walk or wheel to school without their parental supervision. As explained by one parent (child aged 6 −12 years) “*When I was younger, I was always outside. But now the way people are, I have to be outside with [my kids]. If I can’t see them. I’ll run after them. I used to walk to school [but] I’m afraid to have them walk to school. Unless I’m driving behind them"* [60] (Parent, child aged 6 - 12 years). Finally, some parents of children with overweight/obesity described how stigma and bias from their peers and sometimes coaches prevented their child from participating in activities or sports at school or local community. One parent discussed how *“the people organizing the activities don't use your kids in games because they're 'fat' or they're 'slow', and it's adults saying that to kids." * [76] (Parent, child aged 6 - 12 years).

## Discussion

**Fig. 2:**
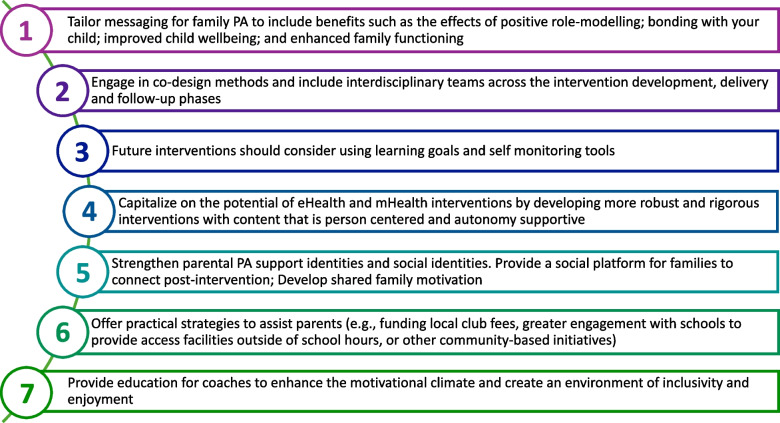
Key recommendations for policy and practice

Despite reports of mixed-effectiveness [22–25], family-based PA interventions are broadly recognized as a necessary approach for improving child and adolescent PA behaviors [9, 21]. This is the first review to appraise and synthesize parents’ experiences of family-based interventions targeting PA in children and adolescents. Our search of the literature retrieved 85 reports representing 82 disparate studies of family-based interventions including children with overweight/obesity, autism, intellectual and/or physical disabilities (e.g., visual impairments, cystic fibrosis) and families of low-SES. The methodological quality of the included studies was assessed using the CASP checklist [41]. We developed 4 analytical and 8 descriptive themes identifying factors that may enhance engagement and mitigate barriers to effective implementation. These themes were developed by synthesising corroborating concepts thus ‘going beyond’ the findings of the original studies [44]. All subgroups (i.e., age-group, illness or disability) are represented in each theme, thus our findings discussed below and summarized in Fig. 2, offer practical recommendations to guide future research, policy and practice that are applicable to a diverse range of families.

### Tailor messaging for family PA

Parents in the review cited the physical and mental health benefits of PA as motives for enrolling in the interventions, reflecting the well-recognized public knowledge of these benefits [80, 81]. However, they also expressed a desire to be a good role model for their children by increasing their PA and improving health related behaviors (e.g., health eating). Many parents shared additional unexpected outcomes for their child that arose from PA engagement, such as greater social skills, enhanced self-concept, improved resilience in challenging situations, and increased school attendance. Remarkably, none of the parents in our review mentioned these outcomes as motives for enrolling in the intervention, they were viewed as a welcome by-product of taking part. Thus, these findings present an opportunity for health promoters to tailor their messaging and increase the likelihood of behavioral impact [82, 83].

This synthesis denotes how parents perceived that family cohesion (i.e., emotional bonding and connection between family members) and organization (i.e., roles, structure and alliance formation) improved as a result of taking part in the interventions, a finding in line with a recent review showing that family PA interventions can improve family cohesion and organization, especially when children are of elementary school age (5–12 years) [40]. Family interventions promoting PA provide an opportunity to improve family functioning [40, 84], hence targeting these constructs (i.e., cohesion, organization, communication, affective environment, and problem solving ability) in intervention development and again tailoring messaging to promote their associated benefits during recruitment for family-based PA interventions may be a fruitful approach [82, 83]. With this in mind, an interdisciplinary team including pediatric psychologists and behavioral scientists would need to be involved across the intervention development, delivery and follow-up phases.

### Engage in co-design methods

Parents’ mostly viewed the interventions as beneficial, flexible, not too challenging to adhere to and an enjoyable experience for all the family. There were some suggestions for improvements, mainly highlighting the need for practical strategies to enable families achieve their goals and additional social support post-intervention to assist with sustaining PA and health related behaviors. Notably, none of the interventions in the review appeared to have used co-design methods as part of the intervention development process, which might have addressed some of these needs in advance [27, 85]. Indeed, engaging with key stakeholders (e.g., parents, children, wider family members, coaches) is now recognised as an integral part of the intervention development process, leading to interventions that are more contextually relevant and ultimately more effective in improving health outcomes [85, 86]. Co-design methods enable the specific needs and preferences of the end-users to be recognised and allows for the identification of potential implementation and evaluation problems early in the intervention development process [27, 85], thus future interventions should consider using co-design methods in the intervention development process.

### Use learning goals and self-monitoring tools

The second analytic theme reflected mixed findings regarding how the interventions content, materials and resources influenced parents’ ability to support their child(ren) to be active. Due to its potential effectiveness [24, 87], goal setting is one of the most frequently used behavior change techniques in interventions promoting PA [88, 89], especially for dyads with strong relationship bonds and where members have the same goal for themselves and other person (e.g., parent–child) [90, 91]. Conversely, some parents in our review described challenges associated with translating goals into PA behavior, aligning with research stating that specific challenging goals can act as a barrier to performance in certain circumstances (e.g., 10,000 steps per day) [92, 93]. This is particularly relevant for those who are physically inactive or at the early stages of learning, as they may find these goals too challenging or stressful, thus highlighting the need for a more dynamic approach to goal-setting [92, 94]. To this end, future interventions could consider using learning goals (i.e., strategies, processes or procedures to be developed in order to master a task) at the early stages of learning a new task (e.g., planning strategies to enable active travel to school), whereas performance goals (e.g., 10,000 steps per day) can increasingly be introduced as skills, knowledge, ability and commitment increase [95, 96].

Most parents described how their child(ren) experienced a sense of pride and accomplishment when using the pedometers to monitor and receive feedback on their activity levels, with a few describing challenges associated with accelerometer wear. A recent review examining the effects of wearable activity trackers (i.e., pedometers, accelerometers, smartwatches, wristbands) found that wearables showed a small significant increase daily step counts among children and adolescents with varying health statuses but not for moderate-to-vigorous physical activity [97]. These findings highlight the potential of activity trackers for motivating PA, particularly for interventions that use gamification [98, 99]. However, there a need for more robust and rigorously designed interventions that minimize missing data to validate the positive findings on step count and investigate potential long-term effects [97–99].

### Capitalize on the potential of eHealth and mHealth interventions

Technological advances have seen an increased use of eHealth and mHealth applications as a mode of delivery for promoting PA children and adolescents [100–102] and families [100]. Indeed, mHealth applications were considered acceptable for parents in our review due to their convenience and flexibility, although some parents expressed a desire for a blend of both mHealth/eHealth and face-to-face modes of delivery, aligning with research for a mother-daughter mHealth intervention [103]. The impact of intervention content (e.g., behavior change techniques) on behavior may vary depending on its delivery style, and in doing so influence its overall effectiveness [104]. Many parents in the review perceived the intervention's delivery style as family-centered, supportive and non-judgmental, particularly in interventions that were culturally tailored. Additionally, providing autonomy support (e.g., providing choice) was viewed as important for parents, specifically when it came to offering guidance on parenting practices and strategies for communicating with their child(ren). Self-Determination Theory (SDT) [105] espouses that autonomous motivation for a behavior is formed by the satisfaction of the basic psychological needs of autonomy, relatedness and competence. A growing body of literature suggests that eHealth and mHealth intervention content that is underpinned by the principles of SDT can enhance the digital therapeutic alliance and promote behaviors such as PA [106–108] Thus, future eHealth and mHealth interventions could apply the principles of SDT to intervention content to ensure a delivery style that is person centered and autonomy supportive (e.g., promoting choice, collaborative goal setting; and encouragement).

### Strengthening parental support identities and social identities

Aligning with research [22], our synthesis found that parents’ knowledge and confidence for PA improved as result of the intervention. Interestingly, some mothers in the review described how it was challenging to incorporate their own PA and family co-activity into their busy schedules [109, 110]. Qualitative research with mothers found that maternal PA identity and confidence to be active may facilitate or hinder their intentions towards PA and PA support behaviors, especially co-activity [42, 110]. Thus, given the important role parental support plays in children’s PA behaviors [19, 111], in particular regarding co-activity [112], future interventions should focus on strengthening parental PA and PA support behaviors via social cognitive and/or other approaches that target PA identity [113–116].

Interestingly, the first analytic theme indicated how it was easier for parents to motivate their children to be active when the entire family were engaged in activities together. However, research investigating the role of shared motivations (i.e., intentions, goals, attitudes, beliefs and enjoyment) towards PA within core family systems (i.e., adult partners and/or children within the family home) has largely been overlooked [117]. This is noteworthy, as the concept of shared motivation is likely a precursor to or closely associated with a family's social identity in relation to PA behaviors [118] and social identities play an important role in promoting and sustaining PA behavior [119, 120].

An individual's identity is typically composed of self-described characteristics (i.e., self-identity) and their affiliation with a social group (i.e., social identity) [115]. Research has found that people are more likely to engage in and sustain exercise among the social groups they strongly identify with and that their sense self becomes incorporated within those social groups [119, 120], thus rendering social identities an important driver of human behavior [118, 121]. In relation to family-based interventions providing opportunities for participants to foster and harness social identities could increase the likelihood of intervention enjoyment and support sustained behavior change [120, 121]. This could be achieved by offering a platform (e.g., via Facebook/WhatsApp groups, obesity care, disability groups) for participating families to connect and establish shared goals among a social group with common interests, values and priorities [118]. This is of particular importance for tailored interventions for children with overweight/obesity or illness or disability [119, 121] given the additional barriers to PA participation that these populations experience (e.g., lack of mobility, stigma and bias) [122–124] and the unique challenges parents face regarding parenting for PA for this cohort (e.g., lack of adapted resources in their community) [122, 125].

### Practical strategies to assist parents provide PA support

Finally, our synthesis revealed how competing priorities, financial constraints, safety concerns and the availability of resources in their community affected parents’ decision making around PA, findings that are consistent with a body of research [110, 126, 127], particularly for families of low SES [128, 129] and children with overweight/obesity [124, 125], or disability [123, 130]. Future policy development should consider measures such as; the substitution of local club fees [131], greater engagement with schools to provide “safe” access to facilities outside of school hours [12], or the use of initiatives such as play streets [132] to provide parents with opportunities to support their child(ren)’s PA.

Finally, parents described how other family members disregard for the intervention presented challenges. This finding was more prevalent in families where children spent their time between two different households, due to parental separation or divorce, highlighting the complexity of the wider social environment on parental support for PA and health related behaviors [117, 133]. Future research should consider including wider family members (e.g., stepparents, grandparents, aunts and uncles) and using social network analysis to gain further understanding into the specific configurations, structures, and characteristics of social networks which are connected to children in family-based interventions [134]. Additionally, the role of coaches is another layer that affects parental support for PA [110], for example parents of children with overweight/obesity in our review described how stigma and bias from coaches at their child’s activity acted as a barrier to supporting their child’s engagement in activities. Thus, interventions for coaches to enhance the motivational climate and create an environment of inclusivity and enjoyment may be a useful strategy [135].

### Limitations of methods

Despite the novel findings of this study, the results should be considered within the context of its limitations. Firstly, the scope of our reviewed literature is restricted to published studies and theses, which offer strengths in terms of the underlying quality associated with by the peer review or thesis defense process, as well as the reliability of search accessibility on the topic. However, this focus also presents limitations, particularly due to potential positivity bias and the tendency to avoid null results within the peer review system [136]. As with all qualitative syntheses, this review is dependent on the analysis of secondary data, which was generated under differing theoretical paradigms and methodological approaches [44] and researcher bias influenced how themes were developed, categorized, interpreted and reported [44]. Finally, some studies lacked sufficient detail making it difficult to consider factors like methodological limitations, coherence, adequacy, and relevance. Consequently, a confidence assessment in the qualitative evidence was not conducted using an approach such the GRADE-CERQual [137] thus the credibility of the synthesized findings remains unclear and should be interpreted with caution.

### Limitations of the literature

Overall studies were of mixed quality with only 11 studies covering the 10 items on the CASP [41] checklist. There was a notable absence of the consideration of the relationship between the researchers and the participants and reporting of data analysis methods sufficiently that may have impacted on study research design, data collection, analysis, and the interpretations of the findings within those studies. Despite the inclusion of 82 studies in the synthesis, only one study was conducted in a middle-income country and no studies were from low-income countries, resulting in significant knowledge gaps across these regions. This is unfortunate as the population in low- and middle-income countries (LMICs) represents a key focus for health promotion efforts and the prevention of non-communicable chronic diseases [138], thus high-quality qualitative evaluative studies of family-based interventions in LMIC’s are needed to fill this important gap. Further, we need to be cognisant that the included studies represent experiences of those who have agreed to take part in a family-based intervention and to give feedback. There may be additional barriers or views among those who did not take part, or who dropped out or did not benefit from the intervention. Capturing these perspectives through research studies can be challenging. Nonetheless, it is important to acknowledge that the review findings may portray a more optimistic representation of parents' experiences. Finally, it would be useful to include the voices of the children and adolescents in future syntheses of family-based interventions.

## Conclusions

This is the first review of a disparate body of literature synthesizing parents’ experiences of in family-based interventions targeting PA in children and adolescents. By examining 82 studies we were able to identify the numerous benefits and challenges associated with family-based interventions that are applicable across ages, families, and populations. These findings can be used to by practitioners and policy makers to enhance the development and promotion of more effective and engaging strategies to promote PA in children and adolescents, while strengthening relationships within families and fostering a sense of belonging and inclusiveness for all.

## Supplementary Information

Supplementary Material 1. Prisma checklist.

Supplementary Material 2. ENTREQ guidelines.

Supplementary Material 3. Search terms.

Supplementary Material 4. Individual study characteristics.

Supplementary Material 5. CASP checklist.

Supplementary Material 6. List of contributing studies.

## Data Availability

All data generated or analyzed during this study are included in this published article and its supplementary information files.

## Abbreviations

CASP: Critical Appraisal Skills Programme
PA: Physical activity
QES: Qualitative evidence synthesis
SES: Socioeconomic status
SDT: Self-determination theory
TPB: Theory of planned behavior
WHO: World Health Organization
