# Supplementary material for: Parents’ experiences of family-based physical activity interventions: a systematic review and qualitative evidence synthesis
Source: Int J Behav Nutr Phys Act. 2025 Jul 1;22:90. doi: 10.1186/s12966-025-01778-9 (PMC12220237; doi:10.1186/s12966-025-01778-9)
Supplement: Supplementary file 3 — Supplementary Material 3. Search terms. [file 12966_2025_1778_MOESM3_ESM.docx]

**Table S2: Search Strategies**

**Medline (Ovid):**

1 exp Empirical Research/ or Interviews as Topic/ or Personal Narratives as Topic/ or Focus Groups/ or exp Narration/ or Nursing Methodology Research/ or Narrative Medicine/

2 (Interview or Personal Narrative).pt.

3 interview*.ti,ab,kf.

4 qualitative.ti,ab,kf,jw.

5 (theme* or thematic).ti,ab,kf.

6 ethnological research.ti,ab,kf.

7 ethnograph*.ti,ab,kf.

8 ethnomedicine.ti,ab,kf.

9 ethnonursing.ti,ab,kf.

10 phenomenol*.ti,ab,kf.

11 (grounded adj (theor* or study or studies or research or analys?s)).ti,ab,kf.

12 life stor*.ti,ab,kf.

13 (emic or etic or hermeneutic* or heuristic* or semiotic*).ti,ab,kf.

14 (data adj1 saturat$).ti,ab,kf.

15 participant observ*.ti,ab,kf.

16 (social construct* or postmodern* or post-structural* or post structural* or poststructural* or post modern* or post-modern*).ti,ab,kf.

17 (action research or cooperative inquir* or co operative inquir* or co-operative inquir*).ti,ab,kf.

18 (humanistic or existential or experiential or paradigm*).ti,ab,kf.

19 (field adj (study or studies or research or work)).ti,ab,kf.

20 (human science or social science).ti,ab,kf.

21 biographical method.ti,ab,kf.

22 theoretical sampl*.ti,ab,kf.

23 ((purpos* adj4 sampl*) or (focus adj group*)).ti,ab,kf.

24 (open-ended or narrative* or textual or texts or semi-structured).ti,ab,kf.

25 (life world* or life-world* or conversation analys?s or personal experience* or theoretical saturation).ti,ab,kf.

26 ((lived or life) adj experience*).ti,ab,kf.

27 cluster sampl*.ti,ab,kf.

28 observational method*.ti,ab,kf.

29 content analysis.ti,ab,kf.

30 (constant adj (comparative or comparison)).ti,ab,kf.

31 ((discourse* or discurs*) adj3 analys?s).ti,ab,kf.

32 (heidegger* or colaizzi* or spiegelberg* or merleau* or husserl* or foucault* or ricoeur or glaser*).ti,ab,kf.

33 (van adj manen*).ti,ab,kf.

34 (van adj kaam*).ti,ab,kf.

35 (corbin* adj2 strauss*).ti,ab,kf.

36 child/ or child, preschool/ or Adolescent/ or Students/ or Infant/

37 (preschool child* or toddler or child or children or infant or "really young" or youth* or kid* or boy* or girl*).tw,kf.

38 family support/ or parent-child relations/ or parenting/ or parents/ or fathers/ or mothers/ or single parent/

39 (mother* or father* or parent* or family or families or kinship or kin ship or "parent-child relations").tw,kf.

40 Program Evaluation/ or follow-up studies/ or longitudinal studies/ or interviews as topic/ or "surveys and questionnaires"/ or self report/ or qualitative research/

41 (program* evaluation* or focus group* or parent interview* or exit survey* or follow up or followup or (qualitative adj research) or ((semi-structured or follow-up or followup or exit) adj3 (interview* or survey or questionnaire))).tw,kf.

42 Resistance Training/ or exercise/ or muscle stretching exercises/ or physical conditioning, human/ or running/ or jogging/ or walking/ or dancing/ or sports/ or youth sports/ or "Physical Education and Training"/

43 ("fitness activit*" or exercis* or "physical activit*" or "dance" or "physical exercis*" or "physical training" or "co-physical activit*" or "resistance training" or (resistance adj2 activit*) or fitness or (sport* adj5 (child* or youth* or adolescen* or activit*))).tw,kf.

44 Education/ or early intervention, educational/ or community participation/ or patient participation/ or experimental group/ or Health Promotion/mt or Program evaluation/ or Pilot projects/ or Program development/

45 ((family* or group*) adj2 based adj5 (program* or session or sessions or workshop* or "play kit" or toolkit or intervention* or initiative* or experimental group* or controlled trial)).tw,kf.

46 44 or 45 [intervention]

47 42 or 43 [PA]

48 36 or 37 [kids]

49 38 or 39 [parents]

50 40 or 41 [design]

51 or/1-35 [qualitative]

52 50 or 51 [qualitative design combined]

53 46 and 47 and 48 and 49 and 52

**APA PSYCHINFO:**

1. ( (DE "Program Evaluation" OR DE "Followup Studies" OR DE "Longitudinal Studies" OR DE "Questionnaires" OR DE "Surveys" OR DE "Self-report" OR DE "Qualitative Methods" ) OR TI ( program* evaluation* or focus group* or parent interview* or exit survey* or follow up or followup or (qualitative N research) or ((semi-structured or follow-up or followup or exit) N3 (interview* or survey or questionnaire))) ) OR KW ( program* evaluation* or focus group* or parent interview* or exit survey* or follow up or followup or (qualitative N research) or ((semi-structured or follow-up or followup or exit) N3 (interview* or survey or questionnaire))) ) OR AB( program* evaluation* or focus group* or parent interview* or exit survey* or follow up or followup or (qualitative N research) or ((semi-structured or follow-up or followup or exit) N3 (interview* or survey or questionnaire))) )
2. ( DE "Sports" OR DE "Athletic Training" OR DE "Physical Activity" OR DE "Exercise" OR DE "Running" OR DE "Walking" OR DE "Athletic Participation" OR DE "Dance" ) OR TI ( "fitness activit*" or exercis* or "physical activit*" or "dance" or "physical exercis*" or "physical training" or "co-physical activit*" or "resistance training" or (resistance N2 activit*) or fitness or (sport* N5 (child* or youth* or adolescen* or activit*)) ) AND AB( "fitness activit*" or exercis* or "physical activit*" or "dance" or "physical exercis*" or "physical training" or "co-physical activit*" or "resistance training" or (resistance N2 activit*) or fitness or (sport* N5 (child* or youth* or adolescen* or activit*)) ) OR KW ( program* evaluation* or focus group* or parent interview* or exit survey* or follow up or followup or (qualitative N research) or ((semi-structured or follow-up or followup or exit) N3 (interview* or survey or questionnaire))) )
3. ( ( DE "Education" OR DE "Early Intervention" OR DE "Group Participation" OR DE "Family Intervention" OR DE "Experimental Subjects" OR DE "Health Promotion" OR DE "Program Development") ) ) OR TI (((family or group or community) N3 based) N6 program*) or session or sessions or workshop* or "play kit" or toolkit or intervention* or initiative* or experimental group* or controlled trial) or AB(((family or group or community) N3 based) N6 program*) or session or sessions or workshop* or "play kit" or toolkit or intervention* or initiative* or experimental group* or controlled trial) or KW(((family or group or community) N3 based) N6 program*) or session or sessions or workshop* or "play kit" or toolkit or intervention* or initiative* or experimental group* or controlled trial)
4. ( ( DE "Focus Group" OR DE "Narratives" OR DE "Focus Group Interview" OR DE "Interviews" OR DE "Empirical Methods" OR DE "Qualitative Methods" ) ) OR PT ( interview or personal narrative ) OR AB ( (interview* or qualitative or (theme* or thematic) or ethnological research or ethnograph* or ethnomedicine or ethnonursing or phenomenol* or (grounded N (theor* or study or studies or research or analys?s)) or life stor* or (emic or etic or hermeneutic* or heuristic* or semiotic*) or (data N1 saturat$) or participant observ* or (social construct* or postmodern* or post-structural* or post structural* or poststructural* or post modern* or post-modern*) or (action research or cooperative inquir* or co operative inquir* or co-operative inquir*) or (humanistic or existential or experiential or paradigm*) or (field N (study or studies or research or work)) or (human science or social science) or biographical method or theoretical sampl* or ((purpos* N4 sampl*) or (focus N group*)) or (open-ended or narrative* or textual or texts or semi-structured) or (life world* or life-world* or conversation analys?s or personal experience* or theoretical saturation) or ((lived or life) N experience*) or cluster sampl* or observational method* or content analysis or (constant N (comparative or comparison)) or ((discourse* or discurs*) N3 analys?s) or (heidegger* or colaizzi* or spiegelberg* or merleau* or husserl* or foucault* or ricoeur or glaser*) or (van N manen*) or (van N kaam*) or (corbin* N strauss*)) ) OR TI ( (interview* or qualitative or (theme* or thematic) or ethnological research or ethnograph* or ethnomedicine or ethnonursing or phenomenol* or (grounded N (theor* or study or studies or research or analys?s)) or life stor* or (emic or etic or hermeneutic* or heuristic* or semiotic*) or (data N1 saturat$) or participant observ* or (social construct* or postmodern* or post-structural* or post structural* or poststructural* or post modern* or post-modern*) or (action research or cooperative inquir* or co operative inquir* or co-operative inquir*) or (humanistic or existential or experiential or paradigm*) or (field N (study or studies or research or work)) or (human science or social science) or biographical method or theoretical sampl* or ((purpos* N4 sampl*) or (focus N group*)) or (open-ended or narrative* or textual or texts or semi-structured) or (life world* or life-world* or conversation analys?s or personal experience* or theoretical saturation) or ((lived or life) N experience*) or cluster sampl* or observational method* or content analysis or (constant N (comparative or comparison)) or ((discourse* or discurs*) N3 analys?s) or (heidegger* or colaizzi* or spiegelberg* or merleau* or husserl* or foucault* or ricoeur or glaser*) or (van N manen*) or (van N kaam*) or (corbin* N strauss*)) ) OR KW ( (interview* or qualitative or (theme* or thematic) or ethnological research or ethnograph* or ethnomedicine or ethnonursing or phenomenol* or (grounded N (theor* or study or studies or research or analys?s)) or life stor* or (emic or etic or hermeneutic* or heuristic* or semiotic*) or (data N1 saturat$) or participant observ* or (social construct* or postmodern* or post-structural* or post structural* or poststructural* or post modern* or post-modern*) or (action research or cooperative inquir* or co operative inquir* or co-operative inquir*) or (humanistic or existential or experiential or paradigm*) or (field N (study or studies or research or work)) or (human science or social science) or biographical method or theoretical sampl* or ((purpos* N4 sampl*) or (focus N group*)) or (open-ended or narrative* or textual or texts or semi-structured) or (life world* or life-world* or conversation analys?s or personal experience* or theoretical saturation) or ((lived or life) N experience*) or cluster sampl* or observational method* or content analysis or (constant N (comparative or comparison)) or ((discourse* or discurs*) N3 analys?s) or (heidegger* or colaizzi* or spiegelberg* or merleau* or husserl* or foucault* or ricoeur or glaser*) or (van N manen*) or (van N kaam*) or (corbin* N strauss*)) )
5. ( ( DE "Family" OR DE "Family Relations" OR DE "Family Structure" OR DE "Intergenerational Relations" OR DE "Parenting" OR DE "Parent Child Relations" OR DE "Fathers" OR DE "Mothers" OR DE "Single Parents" ) ) OR TI ( (mother* or father* or parent* or family or families or kinship or kin ship or "parent-child relations") ) OR KW ( (mother* or father* or parent* or family or families or kinship or kin ship or "parent-child relations") )
6. ( ( DE "Child Health" OR DE "Adolescent Health" OR DE "Early Adolescence" OR DE "Late Adolescence" OR DE "Childhood Development" OR DE "Students" ) OR TI ( (preschool child* or toddler or child or children or infant or "really young" or youth* or kid* or boy* or girl* or adolescen*) ) OR KW ( (preschool child* or toddler or child or children or infant or "really young" or youth* or kid* or boy* or girl* or adolescen*) ) OR AB ( (preschool child* or toddler or child or children or infant or "really young" or youth* or kid* or boy* or girl* or adolescen*) )
7. S1 OR S4
8. S2 AND S3 AND S5 AND S6 AND S7

**CINAHL**

1. (MH "Empirical Research") OR (MH "Exit Interviews) OR (MH "Narratives") OR (MH "Narrative Medicine") OR (MH "Focus Groups")
2. PT(Interview OR Personal Narrative) OR (TI(interview* OR qualitative OR theme* OR thematic OR ethnological research OR ethnograph* OR ethnomedicine OR ethnonursing OR phenomenol* OR (grounded N (theor* OR study OR studies OR research OR analys?s)) OR life stor* OR emic OR etic OR hermeneutic* OR heuristic* OR semiotic* OR data N1 saturat$ OR participant observ* OR social construct* OR postmodern* OR post-structural* OR post structural* OR poststructural* OR post modern* OR post-modern* OR action research OR cooperative inquir* OR co operative inquir* OR co-operative inquir* OR humanistic OR existential OR experiential OR paradigm* OR field N (study OR studies OR research OR work) OR human science OR social science OR biographical method OR theoretical sampl* OR (purpos* N4 sampl*) OR (focus N group*) OR open-ended OR narrative* OR textual OR texts OR semi-structured OR life world* OR life-world* OR conversation analys?s OR personal experience* OR theoretical saturation OR ((lived or life) N experience*) OR cluster sampl* OR observational method* OR content analysis OR (constant N (comparative or comparison)) OR ((discourse* OR discurs*) N3 analys?s) OR (heidegger* OR colaizzi* OR spiegelberg* OR merleau* OR husserl* OR foucault* OR ricoeur OR glaser*) OR (van N manen*) OR (van N kaam*) OR (corbin* N2 strauss*)) OR (SU(interview* OR qualitative OR theme* OR thematic OR ethnological research OR ethnograph* OR ethnomedicine OR ethnonursing OR phenomenol* OR (grounded N (theor* OR study OR studies OR research OR analys?s)) OR life stor* OR emic OR etic OR hermeneutic* OR heuristic* OR semiotic* OR data N1 saturat$ OR participant observ* OR social construct* OR postmodern* OR post-structural* OR post structural* OR poststructural* OR post modern* OR post-modern* OR action research OR cooperative inquir* OR co operative inquir* OR co-operative inquir* OR humanistic OR existential OR experiential OR paradigm* OR field N (study OR studies OR research OR work) OR human science OR social science OR biographical method OR theoretical sampl* OR (purpos* N4 sampl*) OR (focus N group*) OR open-ended OR narrative* OR textual OR texts OR semi-structured OR life world* OR life-world* OR conversation analys?s OR personal experience* OR theoretical saturation OR ((lived or life) N experience*) OR cluster sampl* OR observational method* OR content analysis OR (constant N (comparative or comparison)) OR ((discourse* OR discurs*) N3 analys?s) OR (heidegger* OR colaizzi* OR spiegelberg* OR merleau* OR husserl* OR foucault* OR ricoeur OR glaser*) OR (van N manen*) OR (van N kaam*) OR (corbin* N2 strauss*)) OR (AB(interview* OR qualitative OR theme* OR thematic OR ethnological research OR ethnograph* OR ethnomedicine OR ethnonursing OR phenomenol* OR (grounded N (theor* OR study OR studies OR research OR analys?s)) OR life stor* OR emic OR etic OR hermeneutic* OR heuristic* OR semiotic* OR data N1 saturat$ OR participant observ* OR social construct* OR postmodern* OR post-structural* OR post structural* OR poststructural* OR post modern* OR post-modern* OR action research OR cooperative inquir* OR co operative inquir* OR co-operative inquir* OR humanistic OR existential OR experiential OR paradigm* OR field N (study OR studies OR research OR work) OR human science OR social science OR biographical method OR theoretical sampl* OR (purpos* N4 sampl*) OR (focus N group*) OR open-ended OR narrative* OR textual OR texts OR semi-structured OR life world* OR life-world* OR conversation analys?s OR personal experience* OR theoretical saturation OR ((lived or life) N experience*) OR cluster sampl* OR observational method* OR content analysis OR (constant N (comparative or comparison)) OR ((discourse* OR discurs*) N3 analys?s) OR (heidegger* OR colaizzi* OR spiegelberg* OR merleau* OR husserl* OR foucault* OR ricoeur OR glaser*) OR (van N manen*) OR (van N kaam*) OR (corbin* N2 strauss*))
3. (MH "Child") OR (MH "Child, Preschool") OR (MH "Adolescence") OR (MH "Students") OR (MH "Infants")
4. TI ( preschool child* or toddler or child or children or infant or "really young" or youth* or kid* or boy* or girl* ) OR AB ( preschool child* or toddler or child or children or infant or "really young" or youth* or kid* or boy* or girl* )
5. (MH "Family") OR (MH "Parent-Child Relations") OR (MH "Parenting") OR (MH "Fathers") OR (MH "Mothers") OR (MH "Single Parent")
6. TI ( mother* or father* or parent* or family or families or kinship or kin ship or "parent-child relations" ) OR AB (mother* or father* or parent* or family or families or kinship or kin ship or "parent-child relations")
7. (MH "Resistance Training") OR (MH "Muscle Strengthening") OR (MH "Exercise") OR (MH "Athletic Training Programs") OR (MH "Running") OR (MH "Jogging") OR (MH "Walking") OR (MH "Sports") OR (MH "Dancing")
8. TI ( ("fitness activit*" or exercis* or "physical activit*" or "dance" or "physical exercis*" or "physical training" or "co-physical activit*" or "resistance training" or (resistance N2 activit*) or fitness or (sport* N5 (child* or youth* or adolescen* or activit*))) ) OR AB ( ("fitness activit*" or exercis* or "physical activit*" or "dance" or "physical exercis*" or "physical training" or "co-physical activit*" or "resistance training" or (resistance N2 activit*) or fitness or (sport* N5 (child* or youth* or adolescen* or activit*)))
9. (MH "Program Evaluation") OR (MH "Prospective Studies") OR (MH "Exit Interview") OR (MH "Surveys, Questionnaire") OR (MH "Self Report") OR (MH "Qualitative Studies")
10. TI ( (program* evaluation* or focus group* or parent interview* or exit survey* or follow up or followup or (qualitative N research) or ((semi-structured or follow-up or followup or exit) N3 (interview* or survey or questionnaire))) ) OR AB ( (program* evaluation* or focus group* or parent interview* or exit survey* or follow up or followup or (qualitative N research) or ((semi-structured or follow-up or followup or exit) N3 (interview* or survey or questionnaire)))
11. (MH "Education") OR (MH "Early Intervention" OR (MH "Early Childhood Intervention") OR (MH "Experimental Subjects") OR (MH "Health Promotion") OR (MH "Program Development")
12. TI ((family-based or group-based or family-centered) N5 (program* or session or sessions or workshop*) or "play kit" or toolkit or intervention* or initiative* or experimental group*)) ) OR AB((family-based or group-based or family-centered) N5 (program* or session or sessions or workshop*) or "play kit" or toolkit or intervention* or initiative* or experimental group*)))
13. (S1 OR S2)
14. (S1 OR S2) OR (S9 OR S10)
15. (S3 OR S4)
16. (S5 OR S6)
17. (S7 OR S8)
18. (S11 OR S12)
19. (S14 AND S15 AND S16 AND S17 AND S18)

**SPORTDiscus**

1. (((DE "CHILDREN") AND (DE "HEALTH education (Preschool)" OR DE "HEALTH education (Elementary)" OR DE "HEALTH education (Middle school)")) OR (DE "ADOLESCENT health")) OR (DE "SCHOOL children")) OR (preschool child* or toddler or child or children or infant or "really young" or youth* or kid* or boy* or girl*)
2. DE "FAMILY attitudes" OR (mother* or father* or parent* or family or families or kinship or kin ship or "parent-child relations")
3. DE "PHYSICAL training & conditioning" OR DE "Running" OR DE "Jogging" OR DE "Walking" OR DE "Sports" OR DE "Sport for Youth" OR DE "Dancing" OR (fitness or fitness activit* or exercis* program* or dance or exercis* activit* or wellness program* or physical activit* program or sport* program* or "physical exercis*" or "moderate-vigorous physical activit*" or co-physical activit* or resistance training or (resistance N program*) or (sport N2 (child* or youth* or adolescen*))
4. DE "Education" OR DE "Motivational Interviewing" OR DE "Participation" OR "HEALTH promotion" OR (session* or program* or intervention* or workshop*)
5. (parent interview* or exit survey* or (qualitative N research) or ((semi-structured or follow-up or followup or exit) N3 (interview* or survey or questionnaire)
6. (Interview OR Personal Narrative) OR (interview* OR qualitative OR theme* OR thematic OR ethnological research OR ethnograph* OR ethnomedicine OR ethnonursing OR phenomenol* OR (grounded N (theor* OR study OR studies OR research OR analys?s)) OR life stor* OR emic OR etic OR hermeneutic* OR heuristic* OR semiotic* OR data N1 saturat$ OR participant observ* OR social construct* OR postmodern* OR post-structural* oOR post structural* OR poststructural* OR post modern* OR post-modern* OR action research OR cooperative inquir* OR co operative inquir* OR co-operative inquir* OR humanistic OR existential OR experiential OR paradigm* OR field N (study OR studies OR research OR work) OR human science OR social science OR biographical method OR theoretical sampl* OR (purpos* N4 sampl*) OR (focus N group*) OR open-ended OR narrative* OR textual OR texts OR semi-structured OR life world* OR life-world* OR conversation analys?s OR personal experience* OR theoretical saturation OR ((lived or life) N experience*) OR cluster sampl* OR observational method* OR content analysis OR (constant N (comparative or comparison)) OR ((discourse* OR discurs*) N3 analys?s) OR (heidegger* OR colaizzi* OR spiegelberg* OR merleau* OR husserl* OR foucault* OR ricoeur OR glaser*) OR (van N manen*) OR (van N kaam*) OR (corbin* N2 strauss*))
7. S5 OR S6
8. S1 AND S2 AND S3 AND S4 AND S7

**Web of Science**

1. preschool child* or toddler or child or children or infant or "really young" or youth* or kid* or boy* or girl* or "young people" (Topic)
2. TS=(mom* or dad* or mother* or father* or parent* or family or families or kinship or kin ship or "parent-child relations" or family relationships)
3. TS=(program* evaluation* or focus group* or parent interview* or exit survey* or self-reported or follow up or followup or (qualitative NEAR research) or ((semi-structured or follow-up or followup or exit) NEAR/3 (interview* or survey or questionnaire)))
4. TS=("fitness activit*" or exercis* or "physical activit*" or "dance" or "physical exercis*" or "physical training" or "co-physical activit*" or "resistance training" or (resistance NEAR/2 activit*) or "fitness" or "fundamental movement skills" or (sport* NEAR/5 (child* or youth* or adolescen* or activit*)))'
5. TS=(family-based or group-based or program* or session or sessions or workshop* or "play kit" or toolkit or intervention* or initiative* or experimental group* or controlled trial)
6. TS=(interview* OR "qualitative" OR theme* OR thematic OR ethnological research OR ethnograph* OR ethnomedicine OR ethnonursing OR phenomenol* OR (grounded NEAR (theor* OR study OR studies OR research OR analys?s)) OR life stor* OR emic OR etic OR hermeneutic* OR heuristic* OR semiotic* OR data NEAR/1 saturat$ OR participant observ* OR social construct* OR postmodern* OR post-structural* oOR post structural* OR poststructural* OR post modern* OR post-modern* OR action research OR cooperative inquir* OR co operative inquir* OR co-operative inquir* OR humanistic OR existential OR experiential OR paradigm* OR field NEAR (study OR studies OR research OR work) OR human science OR social science OR biographical method OR theoretical sampl* OR (purpos* NEAR/4 sampl*) OR (focus NEAR group*) OR open-ended OR narrative* OR textual OR texts OR semi-structured OR life world* OR life-world* OR conversation analys?s OR personal experience* OR theoretical saturation OR ((lived or life) NEAR experience*) OR cluster sampl* OR observational method* OR content analysis OR (constant NEAR (comparative or comparison)) OR ((discourse* OR discurs*) NEAR/3 analys?s) OR (heidegger* OR colaizzi* OR spiegelberg* OR merleau* OR husserl* OR foucault* OR ricoeur OR glaser*) OR (van NEAR manen*) OR (van NEAR kaam*) OR (corbin* **NEAR/2 strauss*) OR randomized controlled trial* OR Interview OR Personal Narrative)**
7. 1 AND 2 AND 3 AND 4 AND 5 AND 6

**Limitors**: English, French, and Chinese

**Scopus**

1. (TITLE-ABS("interview*" OR "qualitative" OR "theme*" OR "thematic" OR "ethnological research" OR "ethnograph*" OR "ethnomedicine" OR "ethnonursing" OR "phenomenol*" OR ("grounded" W/ ("theor*" OR "study" OR "studies" OR "research" OR "analysis" OR "analyses")) OR "life stor*" OR "emic" OR "etic" OR "hermeneutic*" OR "heuristic*" OR "semiotic*" OR "data" W/1 "saturation" OR "participant observ*" OR "social construct*" OR "postmodern*" OR "post-structural*" OR "post structural*" OR "poststructural*" OR "post modern*" OR "post-modern*" OR "action research" OR "cooperative inquir*" OR "co operative inquir*" OR "co-operative inquir*" OR "humanistic" OR "existential" OR "experiential" OR "paradigm*" OR "field" W/ ("study" OR "studies" OR "research" OR "work") OR "human science" OR "social science" OR "biographical method" OR "theoretical sampl*" OR ("purpos*" W/4 "sampl*") OR ("focus" W/ "group*") OR "open-ended" OR "narrative*" OR "textual" OR "texts" OR "semi-structured" OR "life world*" OR "life-world*" OR "conversation analysis" OR "personal experience*" OR "theoretical saturation" OR (("lived" OR "life") W/ "experience*") OR "cluster sampl*" OR "observational method*" OR "content analysis" OR ("constant" W/ ("comparative" OR "comparison")) OR (("discourse*" OR "discurs*") W/3 "analysis") OR ("heidegger*" OR "colaizzi*" OR "spiegelberg*" OR "merleau*" OR "husserl*" OR "foucault*" OR "ricoeur" OR "glaser*") OR ("van" W/ "manen*") OR ("van" W/ "kaam*") OR ("corbin*" W/2 "strauss*") OR "randomized controlled trial*" OR "Interview" OR "Personal""Narrative") OR AUTHKEY("interview*" OR "qualitative" OR "theme*" OR "thematic" OR "ethnological research" OR "ethnograph*" OR "ethnomedicine" OR "ethnonursing" OR "phenomenol*" OR ("grounded" W/ ("theor*" OR "study" OR "studies" OR "research" OR "analysis" OR "analyses")) OR "life stor*" OR "emic" OR "etic" OR "hermeneutic*" OR "heuristic*" OR "semiotic*" OR "data" W/1 "saturation" OR "participant observ*" OR "social construct*" OR "postmodern*" OR "post-structural*" OR "post structural*" OR "poststructural*" OR "post modern*" OR "post-modern*" OR "action research" OR "cooperative inquir*" OR "co operative inquir*" OR "co-operative inquir*" OR "humanistic" OR "existential" OR "experiential" OR "paradigm*" OR "field" W/ ("study" OR "studies" OR "research" OR "work") OR "human science" OR "social science" OR "biographical method" OR "theoretical sampl*" OR ("purpos*" W/4 "sampl*") OR ("focus" W/ "group*") OR "open-ended" OR "narrative*" OR "textual" OR "texts" OR "semi-structured" OR "life world*" OR "life-world*" OR "conversation analysis" OR "personal experience*" OR "theoretical saturation" OR (("lived" OR "life") W/ "experience*") OR "cluster sampl*" OR "observational method*" OR "content analysis" OR ("constant" W/ ("comparative" OR "comparison")) OR (("discourse*" OR "discurs*") W/3 "analysis") OR ("heidegger*" OR "colaizzi*" OR "spiegelberg*" OR "merleau*" OR "husserl*" OR "foucault*" OR "ricoeur" OR "glaser*") OR ("van" W/ "manen*") OR ("van" W/ "kaam*") OR ("corbin*" W/2 "strauss*") OR "randomized controlled trial*" OR "Interview" OR "Personal""Narrative")OR
2. TITLE-ABS("program* evaluation*" OR "focus group*" OR "interview*" OR "exit survey*" OR "self-reported" OR "follow up" OR "followup" OR ("qualitative" W/ "research" ) OR (("semi-structured" OR "follow-up" OR "followup" OR "exit" ) W/3 ( "interview*" OR "survey" OR "questionnaire" ))) OR AUTHKEY("program* evaluation*" OR "focus group*" OR "interview*" OR "exit survey*" OR "self-reported" OR "follow up" OR "followup" OR ("qualitative" W/ "research" ) OR (("semi-structured" OR "follow-up" OR "followup" OR "exit" ) W/3 ( "interview*" OR "survey" OR "questionnaire" ))) AND
3. TITLE-ABS( "preschool child*" OR "toddler" OR "child" OR "children" OR "infant" OR "really young" OR "youth*" OR "kids" OR "kid" OR "boy*" OR "girl*" OR "young people" OR "adolescent" ) OR AUTHKEY( "preschool child*" OR "toddler" OR "child" OR "children" OR "infant" OR "really young" OR "youth*" OR "kids" OR "kid" OR "boy*" OR "girl*" OR "young people" OR "adolescent" ) AND
4. TITLE-ABS((("family based" or "family centered") W/4 "program*") OR "session" OR "sessions" OR "workshop*" OR "play kit" OR "toolkit" OR "intervention*" OR "initiative*" OR "experimental group*" OR "controlled trial") OR AUTHKEY((("family based" or "family centered") W/4 "program*") OR "session" OR "sessions" OR "workshop*" OR "play kit" OR "toolkit" OR "intervention*" OR "initiative*" OR "experimental group*" OR "controlled trial") AND
5. TITLE-ABS("fitness activit*" OR "exercis*" OR "physical activit*" OR "dance" OR "physical exercis*" OR "physical training" OR "co-physical activit*" OR "resistance training" OR ("resistance" W/2 "activit*") OR "fitness" OR "moderate-vigorous" OR "sport*") OR AUTHKEY("fitness activit*" OR "exercis*" OR "physical activit*" OR "dance" OR "physical exercis*" OR "physical training" OR "co-physical activit*" OR "resistance training" OR ("resistance" W/2 "activit*") OR "fitness" OR "moderate-vigorous" OR "sport*") AND
6. TITLE-ABS("mom*" OR "dad*" OR "mother*" OR "father*" OR "parent*" OR "family" OR "families" OR "parent-child relations" OR "family relationships" OR "single parent*") OR AUTHKEY("mom*" OR "dad*" OR "mother*" OR "father*" OR "parent*" OR "family" OR "families" OR "parent-child relations" OR "family relationships" OR "single parent*"))

**ProQuest Dissertations and Theses**

1. (((MAINSUBJECT.EXACT("Family structure") OR MAINSUBJECT.EXACT("Family relations") OR MAINSUBJECT.EXACT("Parent-child relations") OR MAINSUBJECT.EXACT("Parents & parenting") OR MAINSUBJECT.EXACT("Fathers") OR MAINSUBJECT.EXACT("Mothers") OR MAINSUBJECT.EXACT("Single parents")) OR summary(mother* OR father* OR parent* OR family OR families OR kinship OR kin ship OR "parent-child relations")) AND
2. ((MAINSUBJECT.EXACT("Resistance training") OR MAINSUBJECT.EXACT("Exercise") OR MAINSUBJECT.EXACT("Physical conditioning") OR MAINSUBJECT.EXACT("Running") OR MAINSUBJECT.EXACT("Jogging") OR MAINSUBJECT.EXACT("Walking") OR MAINSUBJECT.EXACT("Sports") OR MAINSUBJECT.EXACT("Youth participation") OR MAINSUBJECT.EXACT("Dancing")) OR summary(fitness OR fitness activit* OR exercis* program* OR dance OR exercis* activit* OR wellness program* OR physical activit* program OR sport* program* OR ("physical exercise" OR "physical exercises") OR "moderate-vigorous physical activit*" OR co-physical activit* OR resistance training OR (resistance adj program*) OR (sport adj2 (child* OR youth* OR adolescen*)))) AND
3. ((MAINSUBJECT.EXACT("Preschool children") OR MAINSUBJECT.EXACT("Children and youth") OR MAINSUBJECT.EXACT("Adolescents") OR MAINSUBJECT.EXACT("Students") OR MAINSUBJECT.EXACT("Infants")) OR summary(preschool child* OR toddler OR child OR children OR infant OR "really young" OR youth* OR kid* OR boy* OR girl*)) AND
4. ((MAINSUBJECT.EXACT("Early intervention") OR MAINSUBJECT.EXACT("Community participation")) OR summary("family-based" OR "family-centered" OR session* OR program* OR intervention* OR workshop*)) AND (((MAINSUBJECT.EXACT("Interviews") OR MAINSUBJECT.EXACT("Exit interviews") OR MAINSUBJECT.EXACT("Narratives") OR MAINSUBJECT.EXACT("Focus Groups")) OR summary((Interview OR Personal Narrative) OR (interview* OR qualitative OR theme* OR thematic OR ethnological research OR ethnograph* OR ethnomedicine OR ethnonursing OR phenomenol* OR (grounded NEAR/4 (theor* OR study OR studies OR research OR analys?s)) OR life stor* OR emic OR etic OR hermeneutic* OR heuristic* OR semiotic* OR data NEAR/1 saturat OR participant observ* OR social construct* OR postmodern* OR post-structural* OR post structural* OR poststructural* OR post modern* OR post-modern* OR action research OR cooperative inquir* OR co operative inquir* OR co-operative inquir* OR humanistic OR existential OR experiential OR paradigm* OR field NEAR/4 (study OR studies OR research OR work) OR human science OR social science OR biographical method OR theoretical sampl* OR (purpos* NEAR/4 sampl*) OR (focus NEAR/4 group*) OR open-ended OR narrative* OR textual OR texts OR semi-structured OR life world* OR life-world* OR conversation analys?s OR personal experience* OR theoretical saturation OR ((lived OR life) NEAR/4 experience*) OR cluster sampl* OR observational method* OR content analysis OR (constant NEAR/4 (comparative OR comparison)) OR ((discourse* OR discurs*) NEAR/3 analys?s) OR (heidegger* OR colaizzi* OR spiegelberg* OR merleau* OR husserl* OR foucault* OR ricoeur OR glaser*) OR (van NEAR/4 manen*) OR (van NEAR/4 kaam*) OR (corbin* NEAR/2 strauss*)))) AND
5. ((MAINSUBJECT.EXACT("Longitudinal studies") OR MAINSUBJECT.EXACT("Interviews") OR MAINSUBJECT.EXACT("Surveys") OR MAINSUBJECT.EXACT("Questionnaires") OR MAINSUBJECT.EXACT("Qualitative research")) OR summary(parent interview* OR exit survey* OR (qualitative NEAR/4 research) OR ((semi-structured OR follow-up OR followup OR exit) NEAR/3 (interview* OR survey OR questionnaire)))))) AND
6. (la.exact("ENG" OR "CHI" OR "FRE") AND
7. stype.exact("Dissertations & Theses"))

**Summary table**

| **Data Base** | **No. of Hits** |
| --- | --- |
| Medline Ovid | 1,410 |
| PsychINFO | 1,381 |
| SportDiscus | 604 |
| CINAHL | 2,379 |
| Web of Science | 3,293 |
| Scopus | 2,848 |
| Proquest | 499 |
| **Total** | 12,414 |
