# Supplementary material for: Parents’ experiences of family-based physical activity interventions: a systematic review and qualitative evidence synthesis
Source: Int J Behav Nutr Phys Act. 2025 Jul 1;22:90. doi: 10.1186/s12966-025-01778-9 (PMC12220237; doi:10.1186/s12966-025-01778-9)
Supplement: Supplementary file 6 — Supplementary Material 6. List of contributing studies. [file 12966_2025_1778_MOESM6_ESM.docx]

| **Study number** | **Reference** |
| --- | --- |
| **1** | **Andermo S**, Lidin M, Hellenius ML, Nordenfelt A, Nyberg G. “We were all together”-families’ experiences of the health-promoting programme–A Healthy Generation. BMC Public Health. 2020 Dec;20:1-9. |
| **2** | **Arredondo** **EM,** Morello M, Holub C, Haughton J. Feasibility and preliminary findings of a church-based mother-daughter pilot study promoting physical activity among young Latinas. Family & community health. 2014 Jan 1;37(1):6-18. |
| **3** | **Ashton LM,** Young MD, Pollock ER, Barnes AT, Christensen E, Hansen V, Lloyd A, Morgan PJ. Impact of a Father–Child, Community-Based Healthy Lifestyle Program: Qualitative Perspectives from the Family Unit. Journal of Child and Family Studies. 2023 Oct;32(10):2995-3008. |
| **4** | **Azevedo KJ,** Mendoza S, Fernández M, Haydel KF, Fujimoto M, Tirumalai EC, Robinson TN. Turn off the TV and dance! Participation in culturally tailored health interventions: implications for obesity prevention among Mexican American girls. Ethnicity & disease. 2013;23(4):452. |
| **5** | **Bach M,** Shenoi S, Winger K, Hendriksz T. Healthier together: a pilot study on the implementation of a novel family centered pediatric obesity prevention program. Journal of Osteopathic Medicine. 2021 Apr 21;121(5):513-20. |
| **6** | **Barber, S. E.,** Jackson, C., Hewitt, C., Ainsworth, H. R., Buckley, H., Akhtar, S., ... & Wright, J. (2016). Assessing the feasibility of evaluating and delivering a physical activity intervention for pre-school children: a pilot randomized controlled trial. *Pilot and feasibility studies*, *2*, 1-13 |
| **7** | **Barr-Anderson DJ,** Adams-Wynn AW, Alhassan S, Whitt-Glover MC. Culturally-appropriate, family-and community-based physical activity and healthy eating intervention for African-American middle school-aged girls: A feasibility pilot. Journal of Adolescent and Family Health. 2014;6(2):6. |
| **8** | **Bergström H,** Haggård U, Norman Å, Sundblom E, Schäfer Elinder L, Nyberg G. Factors influencing the implementation of a school-based parental support programme to promote health-related behaviours—interviews with teachers and parents. BMC public health. 2015 Dec;15:1-9. |
| **9** | **Bridge GL,** Willis TA, Evans CEL, Roberts KPJ, Rudolf M. The impact of HENRY on parenting and family lifestyle: Exploratory analysis of the mechanisms for change. Child : care, health & development. 2019;45(6):850-60. |
| **10** | **Burnet, D. L.,** Plaut, A. J., Wolf, S. A., Huo, D., Solomon, M. C., Dekayie, G., ... & Chin, M. H. (2011). Reach-out: a family-based diabetes prevention program for African American youth. *Journal of the National Medical Association*, *103*(3), 269-277. |
| **11** | **Campbell-Voytal KD**, Brogan Hartlieb K, Cunningham PB, Jacques-Tiura AJ, Ellis DA, Jen KL, Naar-King S. African American adolescent-caregiver relationships in a weight loss trial. Journal of child and family studies. 2018 Mar;27:835-42 |
| **12** | **Cason-Wilkerson R,** Goldberg S, Albright K, Allison M, Haemer M. Factors influencing healthy lifestyle changes: a qualitative look at low-income families engaged in treatment for overweight children. Childhood Obesity. 2015;11(2):170-6. |
| **13** | **Cason-Wilkerson R,** Scott SG, Albright K, Haemer M. Exploration of Changes in Low-Income Latino Families' Beliefs about Obesity, Nutrition, and Physical Activity: A Qualitative Post-Intervention Study. Behavioral sciences. 2022;12(3):73. |
| **14** | **Clarke JL,** Griffin TL, Lancashire ER, Adab P, Parry JM, Pallan MJ. Parent and child perceptions of school-based obesity prevention in England: a qualitative study. BMC public health. 2015;15(1):1224- |
| **15** | **Coleman KJ,** Ocana LL, Walker C, Araujo RA, Gutierrez V, Shordon M, Oratowski-Coleman J, Philis-Tsimikas A. Outcomes from a culturally tailored diabetes prevention program in Hispanic families from a low-income school. The Diabetes Educator. 2010 Sep;36(5):784-92. |
| **16** | **Columna L,** Haegele JA, Barry A, Prieto L. “I Can Do It”: Perceived Competence of Parents of Autistic Children After Participating in a Physical Activity Intervention. Journal of Physical Activity and Health. 2024 Jan 9;1(aop):1-8. |
| **17** | **Corr M,** McMullen J, Morgan PJ, Barnes A, Murtagh EM. Supporting Our Lifelong Engagement: Mothers and Teens Exercising (SOLE MATES); a feasibility trial. Women Health. 2020;60(6):618-35. |
| **18** | **Eg M,** Frederiksen K, Vamosi M, Lorentzen V. How family interactions about lifestyle changes affect adolescents' possibilities for maintaining weight loss after a weight‐loss intervention: a longitudinal qualitative interview study. Journal of Advanced Nursing. 2017 Aug;73(8):1924-36. |
| **19** | **Enright G**, Gyani A, Hyun K, Allman-Farinelli M, Innes-Hughes C, Chen L, Redfern J. What Motivates Engagement in a Community-Based Behavior Change Strategy for Overweight Children?. Health Promotion Practice. 2022 Jan;23(1):174-84. |
| **20** | **Foote SJ.** The Effect of the Family Structure on Child Physical Activity Within a Fitness Intervention: A Theoretical Approach: Auburn University; 2017. |
| **21** | **Forrest D,** Lee A, Hegde AV, Dev D, Saum D, McMillan V, Stage VC. Engaging with health programming in head start: Parents’ perspectives. Journal of Early Childhood Research. 2023 Sep;21(3):355-68 |
| **22** | **Fothergill MA,** Gill G, Graham PL. The beautiful game bringing families together: children’s and parents’ experiences of a family football programme. Health promotion international. 2024 Feb 1;39(1):daad183. |
| **23** | **Fraser C,** Lewis K, Manby M. Steps in the Right Direction, Against the Odds, An Evaluation of a Community‐Based Programme Aiming to Reduce Inactivity and Improve Health and Morale in Overweight and Obese School‐Age Children. Children & society. 2012 Mar;26(2):124-37. |
| **24** | **Gridley N.** Parental perceptions of an indoor bouldering programme for toddlers and pre-schoolers in England: An initial exploratory study. Journal of Adventure Education and Outdoor Learning. 2023 Oct 2;23(4):416-32 |
| **25** | **Ha AS**, Zeng T, He Q, Chan CHS, Fung Y, Ng JYY. Acceptability of a parent-focused program for physical literacy development among parents and children during the COVID-19 pandemic: A qualitative study. Frontiers in public health. 2022;10:924434 |
| **26** | **Happ MB,** Hoffman LA, DiVirgilio D, Higgins LW, Orenstein DM. Parent and child perceptions of a self-regulated, home-based exercise program for children with cystic fibrosis. Nursing research. 2013;62(5):305-14. |
| **27** | **Hardt J,** Canfell OJ, Walker JL, Webb KL, Brignano S, Kira K, et al. Healthier together: Implementation and evaluation of a co-designed, culturally tailored childhood obesity community prevention program for Maori and Pacific Islander children and families. Health promotion journal of Australia. 2024;35(3):744-59. |
| **28** | **Harvey SP.** *The results of a home-based physical activity and nutrition program of preschool children and parent perceptions of barriers* (Doctoral dissertation, University of Kansas). 2008 |
| **29** | **Heerman WJ,** Schludnt D, Harris D, Teeters L, Apple R, Barkin SL. Scale-out of a community-based behavioral intervention for childhood obesity: pilot implementation evaluation. BMC public health. 2018;18(1):498- |
| **30** | **Heimendinger J,** Uyeki T, Andhara A, Marshall JA, Scarbro S, Belansky E, Crane L. Coaching process outcomes of a family visit nutrition and physical activity intervention. Health education & behavior. 2007 Feb;34(1):71-89. |
| **31** | **Hernández EA.** A validation study on a Latino childhood overweight program. ProQuest; 2007. |
| **32** | **Hinckson EA,** Dickinson A, Water T, Sands M, Penman L. Physical activity, dietary habits and overall health in overweight and obese children and youth with intellectual disability or autism. Research in developmental disabilities. 2013;34(4):1170-8. |
| **33** | **Hodgson C,** Decker D, O'Connor TM, Hingle M, Gachupin FC. A Qualitative Study on Parenting Practices to Sustain Adolescent Health Behaviors in American Indian Families. International journal of environmental research and public health. 2023;20(21):7015. |
| **34** | **Houghton LJ,** O'Dwyer M, Foweather L, Watson P, Alford S, Knowles ZR. An impact and feasibility evaluation of a six-week (nine hour) active play intervention on fathers’ engagement with their preschool children: A feasibility study. Early Child Development and Care. 2015 Feb 1;185(2):244-66. |
| **35** | **Howie EK,** McManus A, Smith KL, Fenner AA, Straker LM. Practical lessons learned from adolescent and parent experiences immediately and 12 months following a family-based healthy lifestyle intervention. Childhood Obesity. 2016;12(5):401-9. |
| **36** | **Hurtado Choque** **GA,** Rodriguez MR, Soltani D, Baltaci A, Nagao-Sato S, Alvarez de Davila S, Monardez J, Peralta Reyes AO, Reicks M. Mixed-Methods Evaluation of Father Participation in an Adolescent Obesity Prevention Program With Multiple Delivery Methods. Health promotion practice. 2024 Nov;25(6):997-1008. |
| **37** | **Hwang Y,** Boyd M, Naylor PJ, Rhodes RE, Liu S, Moldenhauer R, Li J, Wright C, Buckler EJ, Carson V. Piloting the virtual PLAYshop program: a parent-focused physical literacy intervention for early childhood. Children. 2023 Apr 13;10(4):720. |
| **38** | **Ickes S,** Mahoney E, Roberts A, Dolan C. Parental involvement in a school-based child physical activity and nutrition program in Southeastern United States: a qualitative analysis of parenting capacities. Health promotion practice. 2016 Mar;17(2):285-96. |
| **39** | **Jago R,** Sebire SJ, Turner KM, Bentley GF, Goodred JK, Fox KR, et al. Feasibility trial evaluation of a physical activity and screen-viewing course for parents of 6 to 8 year-old children: Teamplay. The international journal of behavioral nutrition and physical activity. 2013;10(1):31- |
| **40** | **Jinks A,** English S, Coufopoulos A. Evaluation of a family‐centred children's weight management intervention. Health Education. 2013 Feb 15;113(2):88-101. |
| **41** | **Karmali S,** Battram DS, Burke SM, Cramp A, Johnson AM, Mantler T, et al. Perspectives and Impact of a Parent-Child Intervention on Dietary Intake and Physical Activity Behaviours, Parental Motivation, and Parental Body Composition: A Randomized Controlled Trial. International journal of environmental research and public health. 2020;17(18):6822. |
| **42** | **Korom B,** Malloy M, Remmers C, Cevilla M, Dione K, Papanek P, Condit J, Nelson D. “It’s about being healthy”; a novel approach to the socio-ecological model using family perspectives within the Latinx community. BMC Public Health. 2023 Jan 11;23(1):86. |
| **43** | **Korom B,** Malloy M, Remmers C, Welsch E, Cevilla M, Alamillo-Roman Z, et al. "It Takes a Village": Reflections from participants after a Hispanic community-based health promotion program. BMC public health. 2024;24(1):237-. |
| **44** | **Lane C,** Carson V, Morton K, Reno K, Wright C, Predy M, Naylor PJ. A real-world feasibility study of the PLAYshop: A brief intervention to facilitate parent engagement in developing their child’s physical literacy. Pilot and Feasibility Studies. 2021 May 26;7(1):113. |
| **45** | **Lane C,** Naylor P-J, Predy M, Kurtzhals M, Rhodes RE, Morton K, et al. Exploring a parent-focused physical literacy intervention for early childhood: a pragmatic controlled trial of the PLAYshop. BMC public health. 2022;22(1):659-. |
| **46** | **Lisinskiene A, Lochbaum M**. A Qualitative Study Examining Parental Involvement in Youth Sports over a One-Year Intervention Program. International journal of environmental research and public health. 2019;16(19):3563. |
| **47** | **Malek ME,** Andermo S, Nyberg G, Elinder LS, Patterson E, Norman A. Parents' experiences of participating in the Healthy School Start Plus programme - a qualitative study. BMC public health. 2023;23(1):646-. |
| **48** | **Monastra MA,** Bordin J, Wolff CB. LEAP Works! Outcomes of a Family-based Nutrition Education and Physical Activity Promotion Program. California Journal of Health Promotion. 2005 Sep 1;3(3):43-60. |
| **49** | **Moore KG.** An Examination of Childhood Obesity in Mississippi: Exploring the Effectiveness of an Early-Intervention Program and Parental Influence: The University of Mississippi Medical Center; 2013. |
| **50** | **Morrison R,** Reilly JJ, Penpraze V, Westgarth C, Ward DS, Mutrie N, Hutchison P, Young D, McNicol L, Calvert M, Yam PS. Children, parents and pets exercising together (CPET): exploratory randomised controlled trial. BMC Public Health. 2013 Dec;13:1-2. |
| **51** | **Neshteruk CD.** Identifying Opportunities to Improve Parent Involvement in Children’s Physical Activity: The University of North Carolina at Chapel Hill; 2019. |
| **52** | **Norman Å,** Nyberg G, Elinder LS, Berlin A. One size does not fit all–qualitative process evaluation of the Healthy School Start parental support programme to prevent overweight and obesity among children in disadvantaged areas in Sweden. BMC Public Health. 2015 Dec;16:1-1. |
| **53** | **O’Brien A,** McDonald J, Haines J. An approach to improve parent participation: In a childhood obesity prevention program. Canadian Journal of Dietetic Practice and Research. 2013 Sep;74(3):143-5. |
| **54** | **Olvera NN,** Knox B, Scherer R, Maldonado G, Sharma SV, Alastuey L, Bush JA. A healthy lifestyle program for Latino daughters and mothers: the BOUNCE overview and process evaluation. American Journal of Health Education. 2008 Sep 1;39(5):283-95. |
| **55** | **Parry YK,** Abbott S, Ankers MD, Willis L, O'Brien T. Beyond Kayaking – A qualitative investigation of parents and facilitators views regrading an outdoor, activity‐based, multi‐session parenting intervention program. Health & social care in the community. 2022;30(2):529-37 |
| **56** | **Pearce K, Dollman J**. Healthy for life pilot study: a multicomponent school and home based physical activity intervention for disadvantaged children. International journal of environmental research and public health. 2019 Aug;16(16):2935. |
| **57** | **Pearson ES,** Irwin JD, Burke SM, Shapiro S. Parental perspectives of a 4-week family-based lifestyle intervention for children with obesity. Global journal of health science. 2012;5(2):111-22. |
| **58** | **Pinard CA,** Hart MH, Hodgkins Y, Serrano EL, McFerren MM, Estabrooks PA. Smart choices for healthy families: a pilot study for the treatment of childhood obesity in low-income families. Health Education & Behavior. 2012 Aug;39(4):433-45. |
| **59** | **Pollock ER,** Young MD, Lubans DR, Barnes AT, Eather N, Coffey JE, et al. Impact of a Father–Daughter Physical Activity Intervention: An Exploration of Fathers’ Experiences. Journal of child and family studies. 2020;29(12):3609-20. |
| **60** | **Poulsen MN,** Hosterman JF, Wood GC, Cook A, Wright L, Jamieson ST, Naylor A, Lutcher S, Mowery J, Seiler CJ, Welk GJ. Family-based telehealth initiative to improve nutrition and physical activity for children with obesity and its utility during COVID-19: a mixed methods evaluation. Frontiers in Nutrition. 2022 Jul 11;9:932514. |
| **61** | **Prieto LA,** Meera B, Katz H, Hernandez MI, Haegele JA, Columna L. Physical activity of children with visual impairments: intentions and behaviors of parents post intervention. Disability and rehabilitation. 2024;ahead-of-print(ahead-of-print):1-9. |
| **62** | **Ptomey LT,** Gibson CA, Willis EA, Taylor JM, Goetz JR, Sullivan DK, Donnelly JE. Parents' perspective on weight management interventions for adolescents with intellectual and developmental disabilities. Disability and health journal. 2016 Jan 1;9(1):162-6. |
| **63** | **Putter KC,** Jackson B, Thornton AL, Willis CE, Goh KMB, Beauchamp MR, et al. Perceptions of a family-based lifestyle intervention for children with overweight and obesity: a qualitative study on sustainability, self-regulation, and program optimization. BMC public health. 2022;22(1):1-16. |
| **64** | **Ransdell LB,** Dratt J, Kennedy C, O'Neill S, DeVoe D. Daughters and mothers exercising together (DAMET): a 12-week pilot project designed to improve physical self-perception and increase recreational physical activity. Women & health. 2001 Aug 21;33(3-4):113-29. |
| **65** | **Reilly KC,** Briatico D, Irwin JD, Tucker P, Pearson ES, Burke SM. Participants' Perceptions of "CHAMP Families": A Parent-Focused Intervention Targeting Paediatric Overweight and Obesity. International journal of environmental research and public health. 2019;16(12):2171. |
| **66** | **Robertson W.** *An evaluation of ‘Families for Health’: a new family-based intervention for the management of childhood obesity* (Doctoral dissertation, University of Warwick). 2009 |
| **67** | **Ross AB,** Quinlan A, Blanchard CM, Naylor P-J, Warburton DER, Rhodes RE. Benefits and Barriers to Engaging in a Family Physical Activity Intervention: A Qualitative Analysis of Exit Interviews. Journal of child and family studies. 2023;32(6):1708-21. |
| **68** | **Rossi MM.** *Assessment of a pilot nutrition education program for Hispanic youth and their parents* (Master's thesis, Clemson University). 2008 |
| **69** | **Sallinen BJ,** Schaffer S, Woolford SJ. In their own words: learning from families attending a multidisciplinary pediatric weight management program at the YMCA. Childhood obesity. 2013;9(3):200-7. |
| **70** | **Schmied EA,** Chuang E, Madanat H, Moody J, Ibarra L, Ortiz K, et al. A Qualitative Examination of Parent Engagement in a Family-Based Childhood Obesity Program. Health promotion practice. 2018;19(6):905-14. |
| **71** | **Schoeppe S,** Waters K, Salmon J, Williams SL, Power D, Alley S, et al. Experience and Satisfaction with a Family-Based Physical Activity Intervention Using Activity Trackers and Apps: A Qualitative Study. International journal of environmental research and public health. 2023;20(4):3327 |
| **72** | **Siwik V,** Kutob R, Ritenbaugh C, Cruz L, Senf J, Aickin M, et al. Intervention in overweight children improves body mass index (BMI) and physical activity. The Journal of the American Board of Family Medicine. 2013;26(2):126-37. |
| **73** | **St. George SM,** Wilson DK, McDaniel T, Alia KA. Process evaluation of the Project SHINE intervention for African American families: An integrated positive parenting and peer monitoring approach to health promotion. Health promotion practice. 2016 Jul;17(4):557-68. |
| **74** | **Sweeney AM,** Wilson DK, Loncar H, Brown A. Secondary benefits of the families improving together (FIT) for weight loss trial on cognitive and social factors in African American adolescents. International Journal of Behavioral Nutrition and Physical Activity. 2019 Dec;16:1-0. |
| **75** | **Tanenbaum ML,** Addala A, Hanes S, Ritter V, Bishop FK, Cortes AL, Pang E, Hood KK, Maahs DM, Zaharieva DP, 4T Study Group. “It changed everything we do”: A mixed methods study of youth and parent experiences with a pilot exercise education intervention following new diagnosis of type 1 diabetes. Journal of Diabetes and its Complications. 2024 Jan 1;38(1):108651. |
| **76** | **Teufel-Shone NI,** Drummond R, Rawiel U. Developing and adapting a family-based diabetes program at the US-Mexico border. Preventing chronic disease. 2004 Dec 15;2(1):A20. |
| **77** | **Tomayko EJ,** Prince RJ, Cronin KA, Kim K, Parker T, Adams AK. The Healthy Children, Strong Families 2 (HCSF2) Randomized Controlled Trial Improved Healthy Behaviors in American Indian Families with Young Children. Current developments in nutrition. 2019;3(Supplement_2):53-62. |
| **78** | **Twiddy M**, Wilson I, Bryant M, Rudolf M. Lessons learned from a family-focused weight management intervention for obese and overweight children. Public health nutrition. 2012 Jul;15(7):1310-7. |
| **79** | **Watson PM,** Dugdill L, Pickering K, Hargreaves J, Staniford LJ, Owen S, Murphy RC, Knowles ZR, Johnson LJ, Cable NT. Distinguishing factors that influence attendance and behaviour change in family‐based treatment of childhood obesity: A qualitative study. British Journal of Health Psychology. 2021 Feb;26(1):67-89. |
| **80** | **Willis, Claire,** Astrid Nyquist, Reidun Jahnsen, Catherine Elliott, and Anna Ullenhag. "Enabling physical activity participation for children and youth with disabilities following a goal-directed, family-centred intervention." *Research in developmental disabilities* 77 (2018): 30-39. |
| **81** | **Willis CE,** Reid S, Elliott C, Nyquist A, Jahnsen R, Rosenberg M, et al. 'It's important that we learn too': Empowering parents to facilitate participation in physical activity for children and youth with disabilities. Scandinavian journal of occupational therapy. 2019;26(2):135-48. |
| **82** | **Wingo BC,** Yang D, Davis D, Padalabalanarayanan S, Hopson B, Thirumalai M, Rimmer JH. Lessons learned from a blended telephone/e-health platform for caregivers in promoting physical activity and nutrition in children with a mobility disability. Disability and health journal. 2020 Jan 1;13(1):100826. |
| **83** | **Wolcott D.** Changing health behaviors: A case study exploring families' participation in a community-based family-centered healthy lifestyle intervention for overweight/obese children. University of Nebraska at Omaha; 2010. |
| **84** | **Woolford SJ,** Sallinen BJ, Schaffer S, Clark SJ. Eat, play, love: adolescent and parent perceptions of the components of a multidisciplinary weight management program. Clinical pediatrics. 2012 Jul;51(7):678-84. |
| **85** | **Yarimkaya E,** Esentürk OK, İlhan EL, Kurtipek S, Işım AT. Zoom-delivered Physical Activities Can Increase Perceived Physical Activity Level in Children with Autism Spectrum Disorder: a Pilot Study. Journal of developmental and physical disabilities. 2023;35(2):189-207. |
